# In silico identification of selective cyclodecapeptide inhibitors targeting *Plasmodium falciparum* Grp78 chaperone

**DOI:** 10.1007/s10822-026-00907-1

**Published:** 2026-08-01

**Authors:** Wendy Mthembu, Adeshina Odugbemi, Florence Lisa Muzenda, Marina Rautenbach, Tawanda Zininga

**Affiliations:** Department of Biochemistry, Stellenbosch University, Stellenbosch, 7599 South Africa

**Keywords:** Malaria, *Plasmodium falciparum* Grp78/Bip, Substrate binding, Peptide-inhibitors, Gramicidin S, Tyrocidines

## Abstract

The increase in drug resistance by *Plasmodium falciparum (Pf)* remains a major challenge in eradicating malaria. The parasite drug resistance towards first line antimalarial therapy is associated with the parasite response to drug induced endoplasmic reticulum (ER) stress. The ER resident glucose-regulated protein 78 (*Pf*Grp78) has been implicated as an ER stress response sensor. *Pf*Grp78 binds to stressed protein substrates to suppress their misfolding and increase the capacity of the parasite ER to maintain proteostasis for parasite survival under stress. However, there have been limited efforts to target the parasite ER protein folding system as a potential drug target. This study sought to identify peptides that mimic the substrates of *Pf*Grp78, which can be potential inhibitors of *Pf*Grp78. Using the chaperone-substrate relationship, we explored the mechanism of action of cyclodecapeptides, gramicidin S (GS) and tyrocidines (Trcs), which were previously shown to exhibit potent antimalarial activity. In this study, using molecular docking and molecular dynamics simulation predictions, we observed that cyclodecapeptides bind to a unique site, suggesting preferential binding towards the substrate binding domain of *Pf*Grp78 (β-SBD). The predicted binding site comprised the arch and pocket residues Gly^426^ to Thr^446^ and Pro^455^ to Val^457^, respectively. Furthermore, our extensive thermodynamics simulations supported the stable binding of the peptides and unveiled distinct inhibitory mechanisms. Our analysis suggests that the anti-plasmodial cyclodecapeptides, TrcA and GS, are predicted to act by inducing conformational locking, which may restrict the dynamic flexibility essential for the *Pf*Grp78 chaperone cycle. Taken together, our results predict preferential binding of the cyclodecapeptides to *Pf*Grp78 over its parasite cytosolic isoform and the human homologs. This offers promise for more experimental validation towards defining the molecular mechanism of action of these compounds.

**Supplementary Information:**

The online version contains supplementary material available at 10.1007/s10822-026-00907-1.

## Introduction

Protein homeostasis, or proteostasis, is essential for the survival and development of *Plasmodium falciparum*, the protozoan parasite responsible for the most lethal form of malaria [1]. *P. falciparum* endures temperature fluctuations and environmental stress during its lifecycle in both mosquito and human hosts. In order to survive and reproduce, the parasite heavily depends on an efficient proteome quality control system modulated by its chaperone network [2]. One of the key regulators of this process is the Glucose-Regulated Protein of 78 kDa (Grp78), also known as Binding Immunoglobulin Protein (BiP), a member of the Hsp70 family that resides in the endoplasmic reticulum (ER). *P. falciparum*, Grp78 (*Pf*Grp78) is thought to assist in folding nascent polypeptides and directing misfolded proteins for degradation via ER-associated degradation pathways (ERAD) [2, 3].

*Pf*Grp78 remains underexplored compared to its mammalian counterpart, although transcriptomic and proteomic data confirm its consistent expression throughout intraerythrocytic development stages [1, 3, 4]. In other eukaryotes, under normal conditions Grp78 is bound to unfolded protein response (UPR) sensors as a repressor, however, under stress it dissociates, and in turn binds to the stressed proteins that are unfolding in the ER lumen. Simultaneously, the unbound UPR sensors get stimulated to elicit a signalling mechanism for UPR [5, 6]. The UPR is upregulated because of misfolded proteins, which implicates *Pf*Grp78 as an important target, as it activates the ER stress sensors that restore the cellular function. These processes have often been described using canonical UPR signalling present in other eukaryotes [6], where the main UPR sensors are inositol-requiring enzyme 1 (IRE1), protein kinase R (PKR)-like endoplasmic reticulum kinase (PERK) and activating transcription factor 6 (ATF6) [7]. However, the IRE1 and ATF6 homologues have not been identified in the *P. falciparum* genome, which suggests that the parasite lacks some members of the canonical three-pronged UPR signalling pathways [8]. Therefore, the parasite UPR is thought to be mediated by PERK [9] whereby during ER stress, the accumulation of misfolded proteins in the ER causes *Pf*Grp78 to dissociate from the transmembrane PERK, causing phosphorylation of downstream eukaryotic translation initiation factor (eIF2), which further increases the translation of UPR dedicated proteins. In addition, this signalling initiates cell cycle arrest [8], which has been associated with ART resistance. Rocamora et al., [10] reported that artemisinin-resistant parasite demonstrated an upregulation of chaperones such as *Pf*Grp78 in the blood stages of the parasite development. This suggested that *Pf*Grp78 is involved in activating the UPR through its capability to shift between substrates, a resting sensor and unfolding protein in the ER lumen.

Generally, Hsp70s bind hydrophobic patches exposed on unfolded polypeptides through its substrate-binding domain (SBD) mainly through the β sheets (SBDβ) and substrate is closed in place by the α-helical lid (SBDα). These events are allosterically modulated by the nucleotide-binding domain (NBD) which binds and hydrolyses ATP, influencing the SBD substrate affinity [11, 12]. Thus, the ATP-bound form of Grp78 shows low substrate affinity, while ADP-bound Grp78 tightly binds unfolded proteins, making nucleotide cycling central to its chaperone function [13–15]. While mammalian Grp78 has been widely studied for its role in cancer, neurodegeneration, and immunity, far less is known about the mechanistic details of *Pf*Grp78, particularly in terms of its structure–function relationships and how these may differ from its human homolog [16, 17]. Emerging studies suggests that despite Grp78 conservation, *Pf*Grp78 has parasite-specific sequences and motifs that distinguish it from human *Hs*Grp78, making it an attractive drug target with a lower risk of host toxicity [18–20]. Notably, work by Chen et al. has shown that certain ATP-competitive inhibitors selectively bind *Pf*Grp78, impairing its function and leading to parasite death without affecting the host counterpart [3]. This provides compelling evidence for exploiting the ATPase cycle and substrate interactions of *Pf*Grp78 as druggable mechanisms. Moreover, it is plausible that *Pf*Grp78 functions in concert with other ER chaperones such as *Pf*Grp94 (Hsp90) and *Pf*Grp170 (Hsp110), forming a proteostasis network analogous to mammalian systems, but with potentially different client specificity and regulation patterns [19, 21].

The functional significance of *Pf*Grp78 is further highlighted by piggyBac gene function screening studies indicating that it is indispensable for asexual blood-stage development of the parasites [22, 23]. Unlike yeast and human cells, where components of the Hsp40-Hsp70-Hsp90 can be partially redundant under non-stressful conditions [24], *P. falciparum* shows a heightened dependency on *Pf*Grp78 and its co-chaperones under all growth conditions. This suggests evolutionary adaptation of the parasite to its complex and stressful lifecycle. Recent sequence analysis and structural modelling of *Pf*Grp78 show key differences with its homologs in surface-exposed loops and potential phosphorylation sites that may affect co-chaperone binding and substrate specificity [19, 25]. These structural variations in the *Pf*Grp78-SBD have implications for specific substrate-binding by the parasite chaperone which can be exploited to develop selective inhibitors that disrupt the substrate binding.

Many antimicrobial peptides including a group of cyclic antimicrobial peptides are characterised by hydrophobic and aromatic sequence motifs alternating with polar residues which presents them as potential substrates for Hsp70s [26]. These motifs are also found in the antimicrobial tyrothricin peptide complex from *Aneurinibacillus migulanus,* and *Brevibacillus* species (Table 1) [27, 28]. The cyclodecapeptide group in tyrothricin comprise of various tyrocidines (Trcs) [29, 30], all of which have potent nanomolar activity against erythrocytic stages of *P. falciparum* [30, 31]. Another cyclodecapeptide, gramicidin S (GS) from *A. migulanus* [33, 34] shares 50% sequence identity with the tyrocidines (Trcs), as well as exhibiting potent anti-plasmodium activity [30, 32]*.* In addition, GS is a major antimicrobial metabolite in *A. migulanus*, compared to the tyrothricin complex [33, 34]. Although antimicrobial peptides have shown promising activity against parasites, many have low bioavailability and protease lability [35, 36]. However, the Trcs and GS maintain a conformational rigidity and a low susceptibility to proteases due to their cyclic nature, contributing to their potent, long lasting biological activity [34]. The target(s) and mechanism of action(s) of these antimicrobial peptides are yet to be fully elucidated. Therefore, this study reports for the first time, using predictive modelling tools, that the ER localised *Pf*Grp78 can be targeted with Trcs, and GS cyclodecapeptides. In addition, this study predicted the important amino acid motifs that facilitate substrate binding in human and *P. falciparum* Hsp70 homologs. These unique substrate binding motifs are potentially involved in the selectivity of the cyclodecapeptides towards the Hsp70 homologs. Furthermore, molecular dynamics simulations and free energy calculations showed different levels of binding affinities between the Hsp70 homologs and the cyclodecapeptides, which is suggestive of selectivity at the atomic level.

**Table 1:** Cyclic decapeptides produced by *Brevibacillus* spp.

Cyclic Peptides	Sequence	Bioactivity	*P. falciparum* IC_50_ (nM) [24]
Gramicidin S (GS)	cyclo(**Phe-Pro**-Val-Orn-Leu-Phe-Pro-**Val-Orn-Leu**)	Compromises the integrity of lipid bilayer of cytoplasmic membrane leading to cell death [30]. Antimalarial activity in *P. falciparum* trophozoites	1300
Tyrocidine A (TrcA)	cyclo(**Phe-Pro**-Phe-Phe**-Asn-Gln**-**Tyr**-**Val-Orn-Leu**)	It exerts its bioactivity through cell membrane disruption [31] Antimalarial activity in *P. falciparum* trophozoites [32]	0.58
Tyrocidine B (TrcB)	cyclo(**Phe**-**Pro**-Trp-Phe-**Asn**-**Gln**-**Tyr**-**Val**-**Orn**-**Leu**)		61
Tyrocidine C (TrcC)	cyclo(**Phe**-**Pro**-Trp-Trp-**Asn**-**Gln**-**Tyr**-**Val**-**Orn**-**Leu**)		290

Conserved amino acid residues are indicated in bold

## Methods and materials

### Multiple sequence analysis of Hsp70 substrate binding domains

The multiple sequence analysis (MSA) was conducted to identify unique motifs on SBDβ of Hsp70 homologs. Amino acid sequences of the canonical Hsp70s from the *P. falciparum Pf*Grp78 (PlasmoDB accession number PF3D7_0917900), *Pf*Hsp70-1 (PF3D7_0818900), *Pf*Hsp70-3 (PF3D7_1134000) and *Pf*Hsp70-x (PF3D7_0831700) were retrieved from PlasmoDB (https://www.PlasmoDB.org). The amino acid sequences of *Homo sapiens* Hsp70 homologs used were obtained from Uniprot (https://www.uniprot.org) with respective accession numbers HspA1A (P0DMV8), *Hs*Hsp70 (HspA1B, P0DMV9), HspA1L (P34931), HspA2 (P54652), *Hs*Grp78 (HspA5, P11021), HspA6 (P17066), HspA8 (P11142), HspA9 (P38646). The MSA was conducted as previously described [36]. Briefly, amino acid sequences corresponding to the residues in position 407–553 (numbering according to *Pf*Grp78) were submitted for multiple sequence alignment analysis using fast Fourier transform (MAFFT) server (https://mafft.cbrc.jp/alignment/server/index.html). The alignments were visualised and colour coded for identity, similarity and divergence using Boxshade server (https://junli.netlify.app/apps/boxshade). Pairwise sequence identities percentages were calculated on BLAST, using the blastp suite (https://blast.ncbi.nlm.nih.gov/).

### Three-dimensional homology models and structure comparison

To assess structural conservation between parasite and human Hsp70s, the three-dimensional (3D) structures of the Hsp70 SBDβ were superimposed on each other using Schrödinger Maestro software (2022-1). The 3D structures were obtained from AlphaFold 4.0 (www.alphafold.ebi.ac.uk/), with UniProt accession numbers *Pf*Grp78 (Q8I2X4), *Pf*Hsp70-1 (P11144), *Pf*Hsp70-3 (Q8II24), *Pf*Hsp70-x (K7NTP5) and the human homologs using the UniProt accession numbers mentioned previously. The 3D structures were quality checked using Ramachandran plots and superimposed onto *Pf*Grp78 as the reference structure to determine structural conservation.

### Protein structure preparation for virtual screening

In order to determine the mechanistic binding of Hsp70 homologs to the peptide inhibitors a virtual screening was evaluated. The 3D protein structures of Hsp70 homologs, retrieved from AlphaFold were used after pre-processing on Schrödinger Bioluminate protein preparation wizard for molecular docking as previously described [37]. Briefly, the 3D structures were pre-processed by filling in the missing hydrogen atoms, assigning the correct bond orders and generating het states with Epik at pH 7.4 ± 2.0. Epik uses the Hammett and Taft empirical Eq. 1 to predict pKa values of ionizable groups of aromatic and aliphatic amino acids. Optimisation of hydrogen bonds was carried out using Propka, and the energy minimisation was performed using default constraint of 0.3 Å, RMSD and OPLS-2005 force field to find the lowest energy conformation of each protein structure.

Hammet and Taft empirical equation:

1 $$pKa={pKa}^{0 }+CF- \sum_{i}^{1}\rho i \sum_{i}^{1}\sigma i, j$$

where CF is the correction factor, ρ is the sensitivity factor for the reaction to polar effects and σ is the polar substituent constant.

### Active site prediction

The Hsp70 SBDβ were investigated for potential binding sites and druggability using SiteMap prediction tool [38, 39]. A 4 Å grid was positioned around the protein to discover potential ligand binding sites based on size, solvent exposure level, tightness, hydrophobic and hydrophilic nature, and hydrogen bonding capacity. The hydrophilicity was measured by adding an electric field reward (negative) term to the van der Waals energy. While the hydrophobicity was measured by adding an electric field penalty (positive) to the van der Waals energy to generate the site score. These were interpreted numerically using site score, which ranks sites which are druggable. The sitescore is interpreted numerically and is defined by Eq. 2.

2 $$ {\text{SiteScore }} = \, 0.0{\text{733 sqrt }}\left( {\mathrm{n}} \right) \, + \, 0.{\mathrm{6688e}} - 0.{2}0{\text{ p}} $$

where n is the number of site points (capped at 100), *e* is the enclosure score and p is the hydrophilic score (capped at 1.0).

### Receptor grid generation

A receptor grid for all the Hsp70 SBDβ 3D structures was generated using the Schrödinger Receptor Grid generation tool as previously described [37]. The selected atoms were within the active site predicted by SiteMap. The area around the SBD region was selected, and a cubic grid box with a length of 20 Å on all sides was generated. The receptor grid was optimised to be conducive for the binding of peptides, and it was specified to be within the residues between position 350–500 which are in proximity to the Hsp70 SBDβ.

### Ligand generation and preparation

The 3D structures of cyclodecapeptides, TrcA, TrcB, TrcC and GS, were generated using homology modelling as previously described [37]. The 3D structure of the model substrate peptide NRLLT (NR) was obtained from the Protein Data Bank (PDB ID: 4R5I), corresponding to the *Escherichia coli* Hsp70 (DnaK) SBD in complex with the NR peptide [40]. This structure was used as the control in all binding predictions. LigPrep tool was used to prepare the peptides as ligands, to generate their various ionization states, tautomer and stereochemistries in their correct chirality. The OPLS-2005 force field was used for optimisation, which produced the low energy conformer of the ligand. The Epik program was used to generate all possible ionisation states for each ligand at pH 7.0 ± 2.0, and realistic tautomers were retained. Chiral carbon atoms were identified and up to 32 possible conformations of ligands were generated for each ligand.

### Molecular docking analysis

Molecular docking experiments were performed with the Schrödinger Glide program as previously described [37]. The prepared 3D models of the Hsp70 proteins were assigned as the receptors, while the cyclodecapeptides were treated as ligands. The receptor was treated as a rigid structure, while ligands were allowed flexibility for the rotation of the amino acid side chains based on the conformations retrieved from LigPrep. Favourable non-covalent interactions were assigned and ranked accordingly. Subsequently, favourable interactions such as hydrophobic, hydrogen-bonding were recognised, and unfavourable interactions such as steric clashes and electrostatic mismatches were penalised. To visualise the protein–ligand interaction, a distance of 2 Å between the relevant residues of the interacting protein-peptide was kept. Binding kinetics, such as binding affinity, binding sites, and interacting residues, were taken into consideration.

### Molecular dynamics simulations

The interaction stability of the docked protein and peptide inhibitors was validated using molecular dynamics simulations (MDS) as previously described [37]. Briefly, the simulations were carried out using Desmond MDS engine implemented in Maestro V 13.1. The top-ranking receptor-ligand interaction from Glide docking were selected from 20 poses. Using the Desmond system builder module in Schrödinger software, each complex was placed in a solvent box with the TIP3P water model. An orthorhombic box was used with a buffer distance of 10 Å between the solute structure (receptor-ligand complex) and the box boundary. Na^+^ and Cl^−^ ions at a concentration of 0.15 M were added to the system. The temperature of 300 K and pressure of 1.01 bar were maintained throughout the simulation. The MDS were run with a total simulation time of 1000 ns for each complex and Apo protein on the Desmond simulation package of the Schrödinger suite. The Hsp70 family dynamics are dominated by slow, large-amplitude inter-domain motions, each system was simulated as a single continuous 1000 ns trajectory (five- to ten-fold longer than the 100 to 200 ns typically reported for protein–peptide complexes) to sample these slow modes within one equilibrium ensemble. Convergence of each trajectory was assessed from the stationarity of the thermodynamic parameters and from the plateauing of the cumulative (running) averages of the Cα-RMSD and radius of gyration. The simulations were run on the GPU compute nodes of the Lengau Centre for High-Performance Computing (CHPC), Council for Science and Industrial Research (CSIR), South Africa. The binding free energy of Hsp70-peptides from MDS trajectories was calculated using the *thermal_mmgbsa.py* script implemented in the Prime module of the Schrödinger suite. From the 10,000 trajectories of each protein–ligand complex, a uniform step size of 25 frames was applied. The molecular mechanics with generalised Born and surface area solvation (MM-GBSA) method was then used to estimate the binding free energy (ΔG_bind), providing insight into the thermodynamic stability of the complexes post-MDS. In Prime MM-GBSA, the binding free energy is estimated using Eq. 3.

3 $$ \Delta {\mathrm{G}}\_{\text{bind }} = \, \Delta {\mathrm{G}}_{{{\mathrm{complex}}}} - \, \Delta {\mathrm{G}}_{{{\mathrm{ligand}}}} - \, \Delta {\mathrm{G}}_{{{\mathrm{receptor}}}} $$

where ΔG_complex_ is the free energy of the optimized protein–peptide complex, ΔG_ligand_ is the free energy of the optimized peptide, and ΔG_receptor_ is the free energy of the optimized receptor. These free energies are computed using the MM-GBSA energy function Eq. 4.

4 $$ \Delta {\mathrm{G}}_{{{\mathrm{bind}}}} = \, \Delta {\mathrm{E}}_{{{\mathrm{MM}}}} + \, \Delta {\mathrm{G}}_{{{\mathrm{solv}}}} + \, \Delta {\mathrm{G}}_{{{\mathrm{SA}}}} $$

where ΔE_MM_ = change in molecular mechanics energy, accounting for the energy of interactions within the complex, ΔG_solv_ is the solvation free energy calculated via a generalized Born implicit solvent model, and ΔG_SA_ is the surface area-based nonpolar solvation energy.

This MM-GBSA formulation yields an enthalpy-dominated effective binding free energy and does not include an explicit solute configurational-entropy (− TΔS) term; the resulting ΔG_bind values were therefore interpreted as a relative ranking metric for the peptide series rather than as absolute binding affinities.

The conformational dynamics of *Pf*Grp78 in its Apo form and in complex with NR, TrcA, TrcB, TrcC, and GS peptides were analysed through free energy landscape construction and principal component analysis (PCA). To characterize the global structural changes, we used the computed RMSD of protein backbone Cα atoms relative to the minimized starting structure and the radius of gyration (Rg) as a measure of overall compactness. The joint probability distribution of the two metrics was generated through two-dimensional histogram analysis with 100 bins, from which the free energy landscape was derived using Eq. 5.

5 $$ \Delta G = \, - RT{\mathrm{ln}}P $$

where *R* is the molar gas constant (equivalent to the Boltzmann constant k_B_ scaled by Avogadro’s number, *N*_A_), *T* is temperature and *P* is the two-dimensional probability distribution of metrics (RMSD, Rg).

The resulting landscapes were visualized as contour plots with energy minima representing thermodynamically stable states.

For finer-resolution analysis of collective motions, PCA was applied to Cα atomic coordinates after trajectory alignment. The first two principal components (PC1 and PC2), capturing the most significant motions, were analysed further. Kernel density estimation was used to compute the probability density in PC space, converted to free energy using Eq. 5 as above.

### Antimicrobial peptide ADMET analysis

The pharmacokinetic and pharmacodynamic properties of model NR, cyclodecapeptides TrcA, TrcB, TrcC and GS were predicted using Qikprop module (Schrödinger Maestro Suite). The absorption, distribution, metabolism, excretion and toxicity properties were evaluated. These include blood brain barrier penetration (BBB), central nervous system (CNS) permeability profiles, metabolic parameters using cytochrome models. The excretion and toxicity profiles were also predicted. The Lipinski rule of five profiles was explored, namely, molecular mass, dipole moment, solvent accessible surface area (SASA), hydrogen bond donor/acceptor traits. Results were analysed using Qikprop properties and descriptors as previously described [37].

## Results

### Multiple sequence alignment analysis

**Fig. 1:**
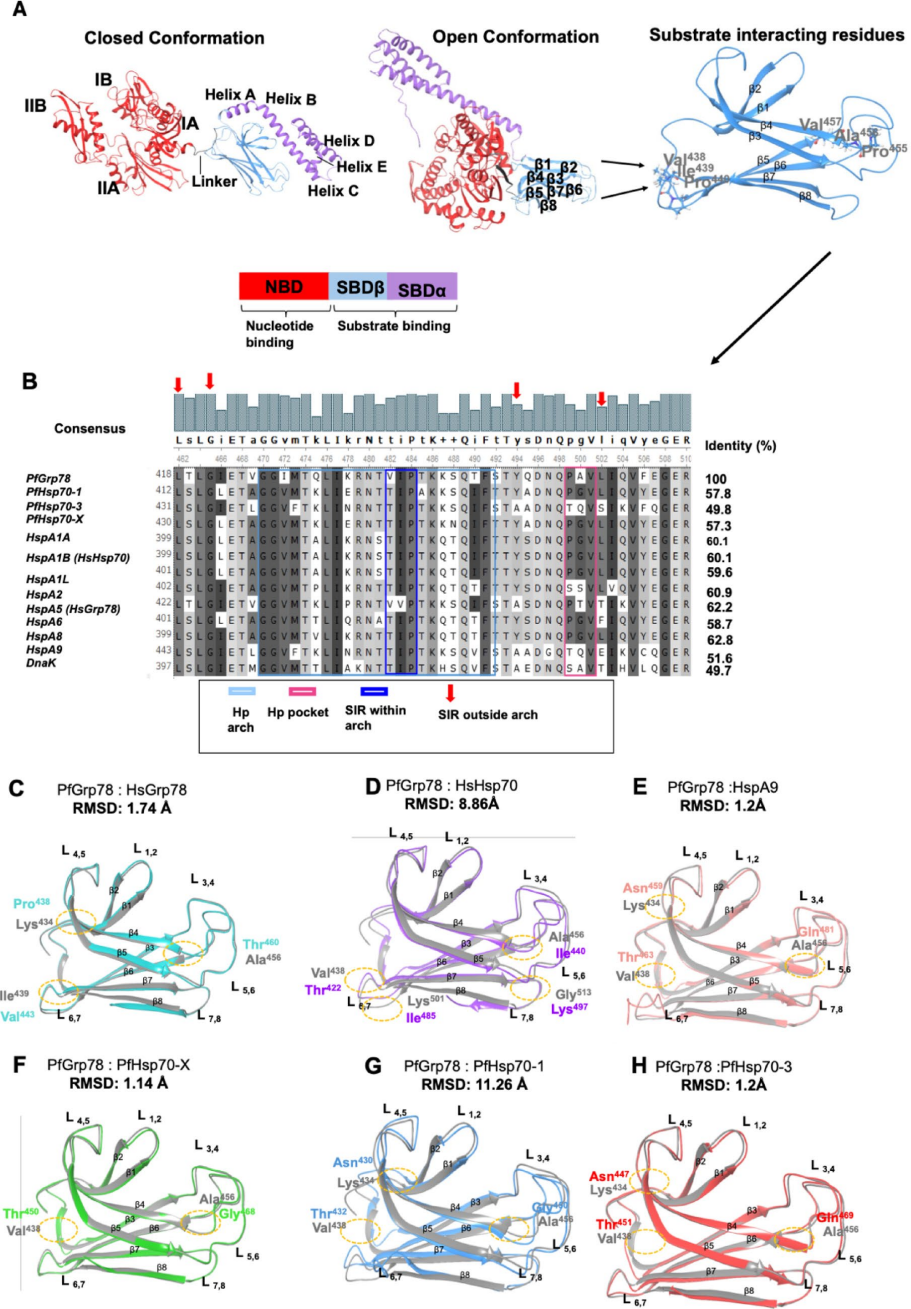
The structural analysis of *Pf*Grp78 β-SBD and comparison with its homologs. **A** Crystal structures of Hsp70 references: 2KHO (closed), 4B9Q (open) with substrate‑binding domain (SBDβ), substrate interacting residues. **B** Multiple sequence alignment of Hsp70 SBDβ, highlights the hydrophobic arch (blue), hydrophobic pocket (pink), and substrate‑interacting residues (SIR; blue), which collectively contribute to substrate‑binding specificity. (Identical residues are indicated in the consensus line, with more identical aa residues indicated in capital letters **C**–**G** Superimposed three‑dimensional structures of *Pf*Grp78 aligned with **C** *Hs*Grp78, **D** *Hs*Hsp70, **E** *Hs*Hsp9A, **F** *Pf*Hsp70‑x, **G** *Pf*Hsp70‑1, and **H** *Pf*Hsp70‑3. These overlays illustrate distinct substrate‑binding motifs and variations in the fold of the hydrophobic arch and pocket across species and cellular compartments. Yellow circles denote regions showing non‑conserved loop motifs that differentiate the Cα‑atom positions among Hsp70 homologs

Multiple sequence analysis of Hsp70s was conducted to determine the level of conservation in the SBDβ. We observed a moderate amino acid sequence identity among the SBDβ of Hsp70 homologs (Fig. 1). The SBDβ of *Pf*Grp78 shares a sequence identity of 58% with its parasite cytosolic isoform *Pf*Hsp70-1, 60% with human cytosolic homolog HspA1B, and 62% with human ER homolog HspA5 (*Hs*Grp78), respectively (Fig. 1A). Sequence conservation is important in predicting the protein function, whereby similarity depicted relatedness of function among the Hsp70 homologs [41]. The substrate interacting hydrophobic arch as defined in [42] at Gly^426^–Tyr^450^ (numbering based on *Pf*Grp78) was conserved at 82% across the Hsp70s. The substrate interacting residues (SIR) within the hydrophobic arch were conserved at 83%. The general Hsp70 SIR possesses a Thr-Ile-Pro motif, but the ER *Pf*Grp78 has a single residue substitution of Val^438^ to yield Val^438^-Ile-Pro^440^ motif, while HspA5 (*Hs*Grp78) showed a Val^442^-Val-Pro^444^ motif. This substitution of a polar uncharged Thr by hydrophobic Val, contributes to the hydrophobicity of the SIR of both parasite and human ER molecular chaperones. Similarly, the substrate binding residues outside the hydrophobic arch Leu^418^ were each conserved in 50% of the Hsp70s, and Thr^421^ was 100% conserved amongst the Hsp70s analysed. In addition, the Hsp70 hydrophobic pocket Pro^455^-Gly^456^-Val^457^ motif was conserved at 58% among cytosolic members across the two species, however, both the parasite ER Grp78 had a Pro-Ala-Val motif where Gly was replaced by a more hydrophobic Ala. Furthermore, the amino acid differences in the hydrophobic pocket and the SIR motifs among the different Hsp70s, although subtle, may be associated with a divergence of function in the SBD as amino acids are essential for direct substrate interaction and allosteric communication with the NBD [43, 44]. These subtle amino acid sequence divergences on important motifs observed here highlight the possibility of selective targeting of the parasite Hsp70s.

### 3D structural analysis of *Pf*Grp78 and its homologs

After analysis of the amino sequence conservation, we explored the 3D structural conservation of *Pf*Grp78 with its Hsp70 homologs. The comparison of the superimposed SBDβ structures shows that there are distinctions between the human and parasite Hsp70s (Fig. 1C-H). The general fold of the Hsp70 backbone carbon-α atoms is similar on the β-sheets hydrophobic cleft in strands β1, β3, β4 which interacts primarily with the hydrophobic groups on substrates [44]. However, *Pf*Grp78 shows a closer structural fold conservation with human Grp78 than with its parasite cytosolic isoform, *Pf*Hsp70-1 (Fig. 1G) and the human equivalent, *Hs*Hsp70 (Fig. 1D) as depicted by higher carbon backbone RMSD greater than 8 Å. In contrast the comparison with the parasite *Pf*Hsp70-x (Fig. 1F), *Pf*Hsp70-3 (Fig. 1H) and the human HspA9 (Fig. 1E) and *Hs*Grp78 (Fig. 1C) had RMSD below 2 Å. These structural comparisons confirmed the differences in folding and orientation on the unique residues of the loops L_1,2_, L_4,5_, L_5,6_, in the substrate binding groove. These were comprised by L_1,2_, Thr^432^ vs Val^438^, between *Pf*Grp78 and *Pf*Hsp70-1 which is polar to non-polar (Fig. 1G). Similarly, L_4,5_ between the parasite and human ER Grp78 was Lys^434^ vs Pro^438^, which is positively charged to non-polar, respectively (Fig. 1C). This suggest that L_4,5_ for parasite does not exhibit similar polar binding with clients but rather non-polar interactions and ionic bonds, setting it apart from human Hsp70s. The L_5,6_ between parasite *Pf*Grp78 and human Hsp70 there was Ala^456^ vs Ile^440^, Gly^513^ vs Lys^497^, (Fig. 1D) and Ala^456^ vs Gly^450^ between *Pf*Grp78 and *Pf*Hsp70-1, respectively (Fig. 1G). This may suggest organelle specialization within the same organism. However, despite high similarities based on RMSD values between the parasite and human ER localised Hsp70, there were differences observed in Lys^434^ (positive charge) vs Pro^438^ (non-polar, structurally rigid), Ile^439^ (non-polar, steric bulk) vs Val^443^ (non-polar, buried) and Ala^456^ (non-polar) vs Thr^460^ (polar), respectively (Fig. 1C). Generally, *Pf*Grp78 and *Hs*Grp78 showed high structural conservation, however, the residues on the loops were distinct, which can contribute to a diverse substrate interaction between them. In addition, the structural conservation was also observed between *Pf*Grp78 with the exported, *Pf*Hsp70-x homolog despite residue differences of Val^438^ vs Thr^450^ and Ala^456^ vs Gly^468^ (Fig. 1F). The mitochondrial isoforms showed structural differences between the β-sheet on Val^438^ vs Thr^451^ and Ala^456^ vs Gly^469^ (Fig. 1H). Taken together, this suggest that there are two potential levels of structural conservation among Hsp70s, either species and/or organelle specificities.

### Structural analysis of the ligands

**Fig. 2:**
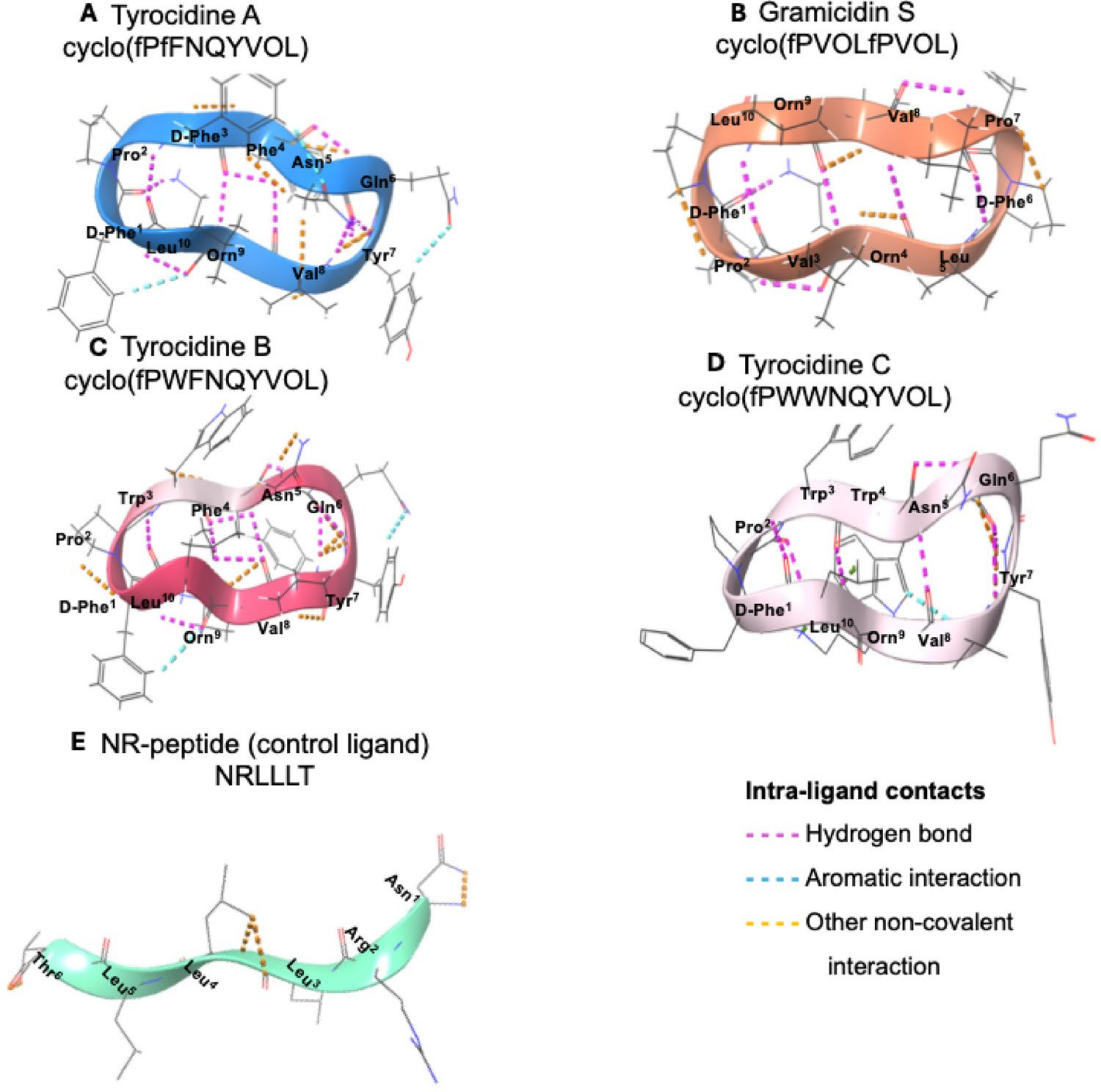
The 3D structures of cyclodecapeptide ligands and the model control peptide. Structural representations of the cyclodecapeptides TrcA, TrcB, TrcC, and GS, alongside the linear model substrate peptide NR. Panels show **A** TrcA, **B** GS, **C** TrcB, **D** TrcC and **E** NR. Stabilising intramolecular contacts including hydrogen bonds, aromatic stacking, and other non‑covalent interactions, are indicated as dotted lines and illustrate the self‑associating interactions that contribute to peptide backbone rigidity and conformational stability

The cyclodecapeptides assessed in this study TrcA, TrcB, TrcC and GS, have been reported to exhibit potent antiplasmodial activity [30, 32] and were analysed for further structural capability in modulating the Hsp70 binding dynamics (Fig. 2). The secondary structure of TrcA reveals an intrapeptide antiparallel β-sheet with a type II β-turn formed by residues (Leu^10^ -D-Phe^1^ -Pro^2^ -Phe^3^) on one side and a distorted type I β-turn on the other side [45, 46] (Fig. 2A). Similarly, the analogues of TrcA, TrcB and TrcC (Fig. 2C, D) retain the same intrapeptide antiparallel β-sheet conformation. While GS is comprised of intrapeptide β-sheet conformation formed by two antiparallel β-strands from the tripeptide sequence (Val-Orn-Leu), interconnected at both ends by two type II β-turns, formed by D-Phe-Pro residues [47, 48] (Fig. 2B). The antiparallel β-sheet of TrcA has a curvature that is distinct from the more planar antiparallel β-sheet of GS (Fig. 2A, B). A known Hsp70s hexapeptide substrate, NR with amino acid sequence Asn-Arg-(Leu)_3_-Thr [40], forms a linear structure (Fig. 1E). The Hsp70 binding on NR is established by the residues (Leu^3^-Leu^4^-Leu^5^) [49–51], and for this study, NR was used as a control for all analyses.

### Protein-peptide molecular docking 

**Fig. 3:**
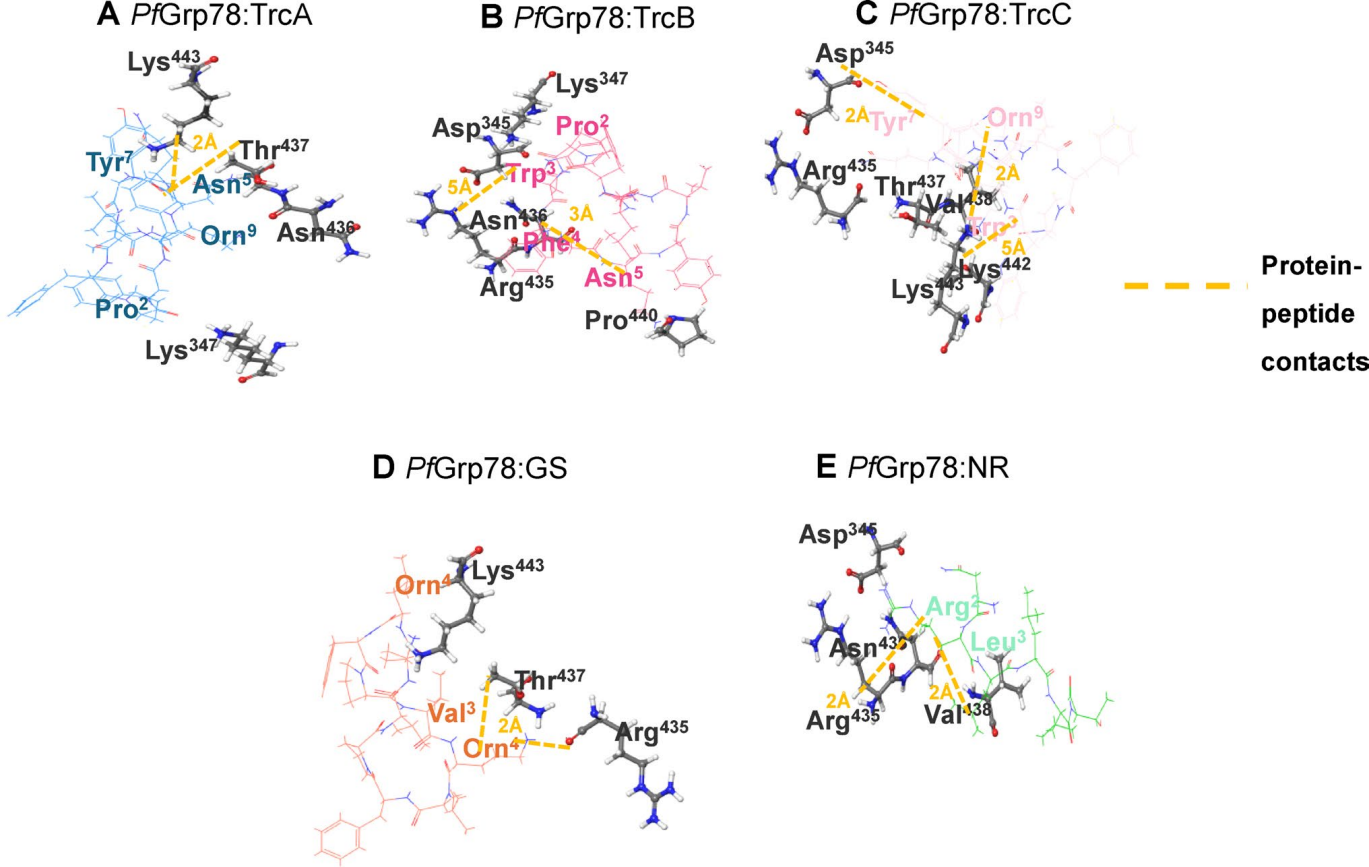
The 3D analysis of *Pf*Grp78 and peptide docking. The figure shows the bond types and interacting residues between *Pf*Grp78 SBD-peptide bound complex. **A** *Pf*Grp78-TrcA, **B**. *Pf*Grp78-TrcB, **C**. *Pf*Grp78-TrcC, **D** *Pf*Grp78-GS, **E** *Pf*Grp78-NR. The nature of the binding interactions was mainly comprised of hydrogen bonds and salt bridges as shown by the coloured lines (yellow) between protein–ligand complexes while the yellow circle highlights where interesting interactions were occurring and the distances between them

The protein-peptide binding was analysed to identify the possible binding modes through Glide molecular docking and showed all the Hsp70s as druggable candidates (Supplementary Table S1). The molecular docking analysis shows that both human and parasite Hsp70s, they all bind to NR peptide, with the lowest docking score of − 9.3 kcal/mol for both *Hs*HspA9 and *Hs*HspA2. In contrast, the highest docking score of NR with HspA5 was − 5.7 kcal/mol (Supplementary Table S2). As a control peptide for canonical Hsp70s, this validated our workflow (Supplementary Figure S1). After this, when comparing the binding of the peptides to the Hsp70s, we observed that, *Pf*Grp78 had capability to bind all peptides with favourable docking scores. Similarly, the mitochondrial *Pf*Hsp70-3 was predicted to bind most of the peptides with favourable docking scores except TrcB at − 3.4 kcal/mol (Supplementary Table S2). Considering this, we then focused on *Pf*Grp78 to explore the special features that enable it to bind all peptides when others were limited. *Pf*Grp78 was predicted to bind the cyclodecapeptides with a lowest docking score of − 7.434 kcal/mol for TrcA (Fig. 3A), followed by TrcC with − 7.232 kcal/mol (Fig. 3C) and − 6.617 kcal/mol for GS (Fig. 3D) and the highest was − 6.389 kcal/mol for TrcB (Fig. 3B Table 2). *Pf*Grp78 was predicted to bind the NR peptide with a favourable docking score in the same range as the cyclodecapeptides peptides of − 7.30 kcal/mol (Fig. 3E, Table 2). This was conceivable considering that Grp78 is a canonical Hsp70 which is close to DnaK. Furthermore, analysis of the interacting residues showed that *Pf*Grp78 bound to NR with similar docking scores to that of TrcA, but it was through different binding amino acid residues.

**Table 2:** Summary of the* Pf*Grp78-peptide docking analysis

Peptide	Peptide residue	SBD residue	Putative type of non-covalent interaction within 2 Å	Distances (Å)	Docking score (kcal/mol)
Trc A	Pro^2^	Lys^347^	H-bond - Pro carbonyl group with Lys side chain amino group	2.09	− 7.434
	Orn^9^	Asn^436^	H-bond - Orn side chain amino group with Asn carbonyl group	1.99	
	Asn^5^	Thr^437^	H-bond - Asn side chain amino group with Thr side chain hydroxyl group	1.79	
	Asn^5^	Lys^443^	H-bond - Asn side chain carbonyl group with Lys side chain amino group	2.23	
TrcB	Trp^3^	Asp^345^	H-bond – Trp side chain aromatic group with Asp side chain	4.16	− 6.389
	Trp^3^	Lys^347^	H-bond -Trp side chain carbonyl group with Lys side chain amino group	5.04	
			Pi-cation Trp side chain aromatic group with Lys side chain amino group	4.58	
	Asn^5^	Arg^435^	H-bond -Asn side chain amide group with Arg side chain group	3.59	
	Asn^5^	Asn^436^	H-bond -Asn side chain amide group with Asn side chain carbonyl group	2.73	
	Tyr^7^	Pro^440^	H-bond -Tyr side chain hydroxyl group with Pro side chain	2.11	
TrcC	Tyr^7^	Asp^345^	H-bond -Tyr side chain hydroxyl group with Asp side chain side chain carbonyl group	1.81	− 7.23
	Gln^6^	Arg^435^	H-bond -Gln side chain amide group with Arg side chain carbonyl group	2.09	
	Orn^9^	Val^438^	H-bond -Orn side chain amino group with Val side chain carbonyl group	2.31	
	Trp^3^	Lys^443^	Pi-cation -Trp side chain aromatic group with Lys side chain amino group	5.03	
G S	Pro^2^	Lys^443^	H-bond - Pro carbonyl with Lys side chain amino group	1.85	− 6.617
	Orn^9^	Thr^437^	H-bond - Orn side chain amino group with Thr side chain hydroxyl group	1.99	
	Orn^9^	Arg^435^	H-bond - Orn side chain amino group with Arg carbonyl group	1.78	
NR peptide	Asn^1^	Tyr^242^	pi-cation interaction of Asn N-terminal amino group and Tyr side chain	1.96	− 7.52
	Asn^1^	Tyr^242^	H-bond - Asn side chain amino group with Tyr carbonyl group	1.87	
	Asn^1^	Glu^240^	Salt bridge - Asn N-terminal amino group with Glu side chain carboxylate	2.48	
	Asn^1^	Val^241^	H-bond - Asn N-terminal amino group with Val carbonyl group	2.18	
	Arg^2^	Asp^345^	H-bond - Arg guanidino group with Asp carbonyl group	1.87	
	Arg^2^	Arg^435^	H-bond - Arg guanidino group with Arg carbonyl group	1.97	
	Arg^2^	Val^438^	H-bond - Arg amino group with Val carbonyl group	1.80	

The analysis of the bonds formed during NR peptide binding to *Pf*Grp78 we observed that they were mainly through hydrogen bonds (H-bonds), salt bridges and pi-cation (Table 2; Supplementary Figure S2). The NR N-terminal dipeptide moiety, Asn^1^-Arg^2^ was responsible for the most stable bonds. The peptide Asn^1^ formed pi-cation and H-bond with the protein Tyr^242^ side chain and the Val^241^ carbonyl group. In addition, Arg^2^ guanidino group formed H -bonds with the carbonyl groups on Arg^345^, Arg^435^ and Val^438^ (Supplementary Figure S2, Table 2). Among the cyclodecapeptides, TrcA, bound to *Pf*Grp78 through four H-bonds between residues Pro^2^ carbonyl group, Orn^4^ side chain amino group and Asn^5^ both amino and carbonyl group with Lys^347^ amino group, Asn^436^ carbonyl group, Thr^437^ hydroxyl group on its side chain and Lys^443^ side chain amino group (Fig. 3A, Table 2). In addition, TrcA mimics the typical Hsp70-substrate. It possesses 5 hydrophobic amino acids (Phe^1,4^, Val^8^, Leu^10^ flanked by a Tyr^7^ which contributed to the high docking score compared to GS. The lower GS binding affinity for *Pf*Grp78 may be due to only three H-bonds from carbonyl group on Pro^2^ and amino group on the side chain of Orn^4^ binding to Lys^443^ amino side chain, Thr^437^ side chain hydroxyl group and Arg^443^ carbonyl group, respectively (Fig. 3D, Table 2). These were different to those observed for the cytosolic human homolog, *Hs*Hsp70 (Supplementary Figure S3), the parasite cytosolic isoform, *Pf*Hsp70-1 (Supplementary Figure S4) and the mitochondrial isoform *Pf*Hsp70-3 (Supplementary Figure S5). This suggests unique substrate interaction among the Hsp70s, which could be exploited for further selective targeting.

To further examine the predicted binding preference of *Pf*Grp78 to the peptides, we observed that the *Pf*Grp78 substrate binding residues Asp^436^ and Thr^437^ (Fig. 1) were shown to both interact with the TrcA while GS interacted only with Thr^437^. Interestingly, *Pf*Grp78 residue Arg^435^ is predicted to be involved in hydrogen bonds with Pro^2^ on GS and Arg^2^ on NR peptide, but not with TrcA. This may suggest that the binding is influenced by other neighbouring residues and not those implicated in TrcA binding such as Lys^347^, Asn^436^, and Lys^443^. To further validate these predicted residue preferences, we exchanged the residues on the *Pf*Grp78 SBD Val^438^-Ile-Pro^440^ motif (Supplementary Table S3 and Figure S6). The residue Val^438^ was shown to be essential in Hsp70 substrate binding, together with Ile^439^ and Pro^440^ they contribute to the hydrophobicity of the pocket (Fig. 1), therefore orienting *Pf*Grp78 for non-covalent binding to substrates [44] and altering the position of the amino acid can modulate the function of the protein. We observed that a change from Val^438^ to substitute it with Lys^438^, or Ile^439^ substituting with Pro^439^, and Pro^440^ substituting it with Ile^440^ resulted in abrogation of the binding to both TrcA and GS.

Similarly, exchanging the arrangement of the whole motif to Leu^438^-Pro-Ile^440^ resulted in no binding predicted. On the contrary, these residue changes did not cause dramatic changes to *Pf*Grp78 docking scores with NR peptide. This is consistent with the predicted preferential binding of TrcA and GS to *Pf*Grp78 over the other Hsp70 homologs, a preference not observed for the model substrate, NR peptide. Taken together, the molecular docking results highlighted the differences among Hsp70 homologs in binding peptides, notably between *Pf*Grp78 and *Hs*Grp78 or its cytosolic isoform *Hs*Hsp70. These results suggest a predicted preferential binding of TrcA and GS to *Pf*Grp78 versus the human homologs. Previously, *Hs*Grp78 was found to preferentially bind hydrophobic domains containing alternating aromatic and hydrophobic amino acid [52]. Conversely, *Pf*Grp78 was found to bind to hydrophobic residues and not to aromatic residues. The essential substrate binding region of *Pf*Grp78 was shown to exhibit binding with cyclodecapeptides within the predicted hydrophobic pocket, but mostly with different polar amino acid residues than those described previously [44].

### Molecular dynamic simulations

**Fig. 4:**
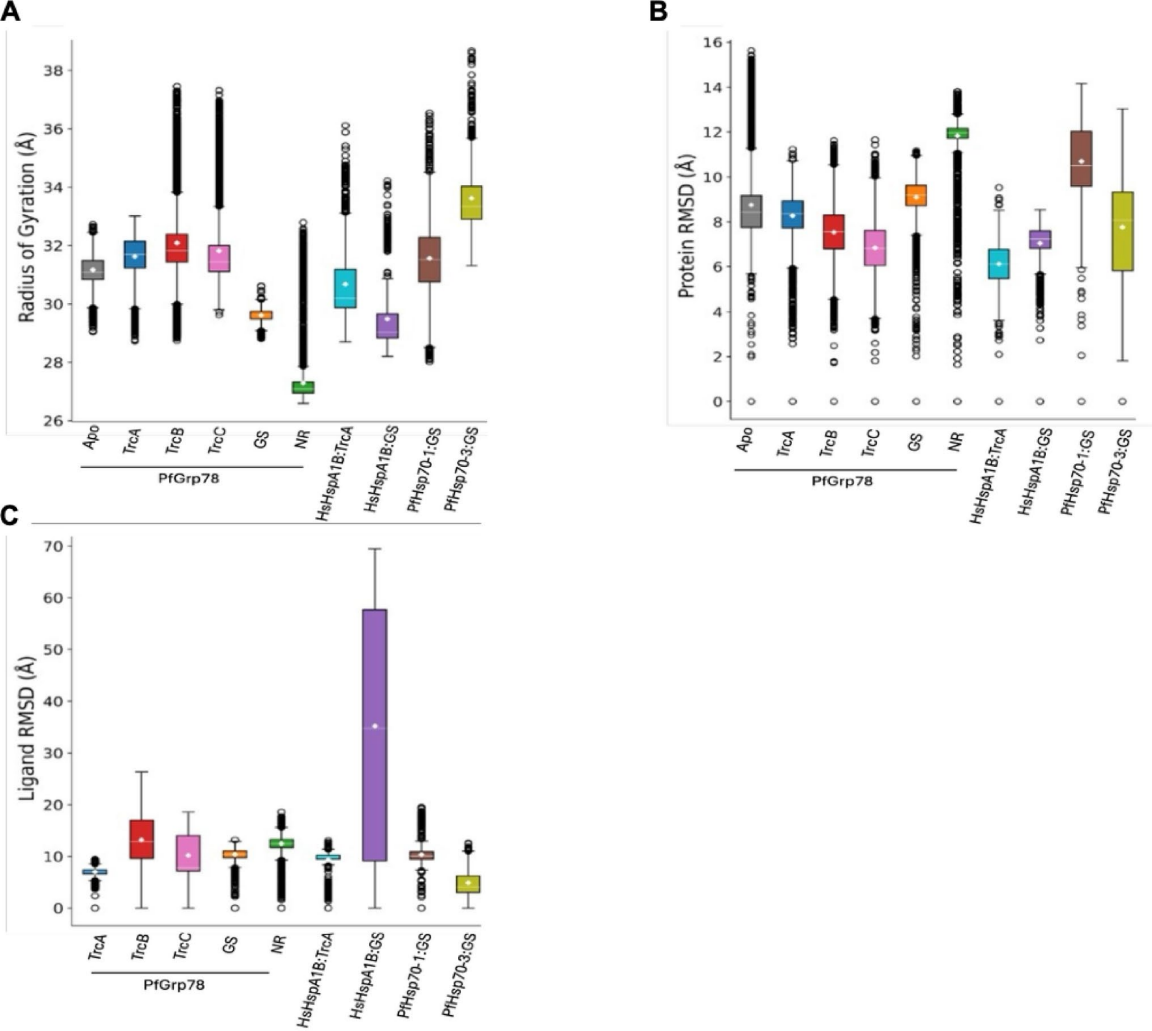
The Box plot of *Pf*Grp78-ligand stabilities during MD simulation. **A** The Rg of Cα atoms. **B** The RMSD plot of *Pf*Grp78 Cα atoms over a simulation period of 1000 ns. **C** RMSD of ligands with respect to protein shows the stability of the peptides with respect to the *Pf*Grp78-binding site. *Hs*Hsp70 is represented as *Hs*HspA1B

To investigate the behaviour and motion of the predicted *Pf*Grp78 docking complexes with the peptides, MD simulations were used. The simulation quality analysis algorithm in Desmond showed that the changes in temperature, energy, pressure and volume during the simulation period were equilibrated for the 1000 ns time scale (Supplementary Table S4, S5). Consistent with this, the Cα-RMSD and radius of gyration reached and maintained stable plateaus (Supplementary Figure S7), and their cumulative averages converged within the trajectory (Supplementary Figure S8), confirming that the systems were properly equilibrated. Furthermore, the Rg, RMSD and root mean square fluctuation (RMSF) parameters were evaluated for Hsp70 homologs that demonstrated binding to peptides (Supplementary Figure S7–S11). The radius of gyration depicts the compactness of *Pf*Grp78 structure in the absence and presence of ligands during the simulation period (Fig. 4A, Supplementary Figure S7A). A lower Rg number indicates that the protein structure is compact, whereas a higher Rg value indicates that it is less compact [48]. A higher Rg value was noted for *Pf*Grp78-Apo (31.16 ± 0.53 Å), *Pf*Grp78-TrcA (31.63 ± 0.66 Å), *Pf*Grp78-TrcB (32.09 ± 1.28) and TrcC (31.82 ± 1.20), which indicates the protein–ligand complex is less compact (Table 3). While *Pf*Grp78-GS and *Pf*Grp78-NR peptide had a more compact structure, indicated by a lower Rg value of (29.61 ± 0.20 Å) and (27.28 ± 0.76 Å), respectively. The human Hsp70 in complex with ligands, *Hs*Hsp70-TrcA (30.68 ± 1.30) and *Hs*Hsp70-GS (29.48 ± 1.30) showed a lower Rg than that of the parasite Hsp70s (Supplementary Table S11). As suggested by Tran et al., [53], a compound that significantly affects the functional mechanism of a protein results in the protein structure becoming less compact. Radius of gyration is a global descriptor of compactness, we interpret these differences only as indicating that the Trc-bound complexes sample, on average, a less compact conformational ensemble than the GS- and NR-bound complexes over the trajectory, and Rg alone does not establish a change in folding stability or chaperone function. Considered together with the free-energy-landscape, PCA (Fig. 7) and structural-superposition analyses (Supplementary Figure S8), this reduced compactness is consistent with, but not direct evidence of, restricted access to the compact states associated with the chaperone cycle. This is a possibility that would require experimental validation. We note that each *Pf*Grp78 system was sampled as a single extended trajectory (1000 ns) rather than as multiple independent replicas; although the convergence diagnostics support the reliability of the reported ensembles, future work incorporating independent replicas would strengthen the statistical robustness of the comparisons.

**Table 3:** Summary of the molecular dynamic parameters of *Pf*Grp78 and peptides

Receptor-ligand complex	Radius of gyration (Å) ± SD	Cα Root mean square deviation (Å) ± SD	Ligand RMSD (Å) ± SD	ΔG_bind average (Kcal/mol) ± SD
*Pf*Grp78-TrcA	31.63 ± 0.66	8.28 ± 0.98	6.95 ± 0.62	− 68.82 ± 9.43
*Pf*Grp78-TrcB	32.09 ± 1.28	7.54 ± 1.21	13.17 ± 4.69	− 19.48 ± 9.17
*Pf*Grp78-TrcC	31.82 ± 1.20	6.85 ± 1.13	10.20 ± 3.62	− 26.64 ± 12.38
*Pf*Grp78-GS	29.61 ± 0.20	9.10 ± 0.87	10.38 ± 0.94	− 64.44 ± 11.75
*Pf*Grp78-NR	27.28 ± 0.76	11.83 ± 0.81	12.44 ± 1.41	− 34.21 ± 7.74
*Pf*Grp78_Apo	31.16 ± 0.53	8.76 ± 1.69	N/A	N/A

The structure variation was calculated by RMSD value of protein–ligand complexes (Fig. 4B, Supplementary S7B). The RMSD indicates how much the protein–ligand conformation has changed overtime when a dynamical system fluctuates. The Cα RMSD of *Pf*Grp78-NR complex (11.83 ± 0.81 Å) and *Pf*Grp78-GS complex (9.10 ± 0.87 Å) were steadily increased when compared to the *Pf*Grp78-Apo (8.76 ± 1.69 Å), *Pf*Grp78-TrcA (8.28 ± 0.98 Å), TrcB (7.54 ± 1.21 Å) and TrcC being the lowest at (6.85 ±1.13 Å) (Table 3). Although these RMSDs exceed the ~3 Å threshold typical of small, globular proteins [54], they are expected for the full-length, two-domain *Pf*Grp78 used here, in which the global Cα-RMSD is dominated by large-scale rigid-body motion of the nucleotide- and substrate-binding domains and the α-helical lid intrinsic to the Hsp70 allosteric cycle, rather than by local structural instability. Consistent with this, the cumulative averages of the RMSD and radius of gyration converged to stable plateaus within the trajectory (Supplementary Figure S8), indicating that these elevated values reflect converged sampling of a mobile multidomain chaperone. Notably, the highest RMSD of *Pf*Grp78-NR complex suggest that the protein underwent a significant but consistent conformational shift, potentially indicating a stable binding-induced rearrangement reducing structural fluctuations [55]. In contrast, the Apo, and Trcs systems exhibited lower RMSD values indicating that their conformations remained relatively close to the initial structure throughout the simulation, possibly in the open conformation. This suggests better structural stability in the absence of the NR peptide or when bound to GS and TrcA. In addition, when *Pf*Grp78 is in complex with TrcB and TrcC there could be further stabilization of the structural conformation as lowest RMSD were observed. The comparatively modest deviations from the Apo state in these systems may reflect less disruptive interactions with *Pf*Grp78.

The ligand RMSD values reveal how stable a ligand is in relation to the protein-binding pocket and relative to the protein indicate the degree to which each peptide-maintained proximity to the binding pocket throughout the simulation. TrcA exhibited the lowest ligand RMSD value (6.95 ± 0.62 Å), suggesting it remained closely associated with the binding site and thus maintained the most stable pocket engagement (Fig. 4C, Table 3 and Supplementary S7). GS and TrcC showed an RMSD (10.2 ± 3.94 Å), reflecting moderate deviation from the binding pocket over time. NR peptide had second from highest RMSD (12.44 ± 1.41 Å), whilst TrcB had the highest RMSD (13.17± 4.69) indicating frequent displacement from the binding region and a more transient binding mode. Taken together, this suggest *P*fGrp78 bound to NR and TrcB more loosely than to TrcA which remained bound for a longer duration. This is consistent with the behaviour of Hsp70s to bind to substrates transiently [50, 56] and in this case NR and the potential inhibitor TrcB.

In addition to the MD simulations on *Pf*Grp78, the stability of ligand binding on *Hs*Hsp70 was also evaluated. The results revealed that TrcA showed a relatively more stable binding to *Hs*Hsp70 compared to GS, although most of its interactions occurred outside the key substrate binding residues. GS binding to *Hs*Hsp70 was not stable, as indicated by a significant increase in the ligand RMSD shortly after 20 ns (Supplementary Figure S10). This is consistent with computationally predicted preferential binding of the cyclodecapeptides to *Pf*Grp78 relative to *Hs*Hsp70 under the conditions simulated; however, experimental validation would be required to confirm true selectivity.

**Fig. 5:**
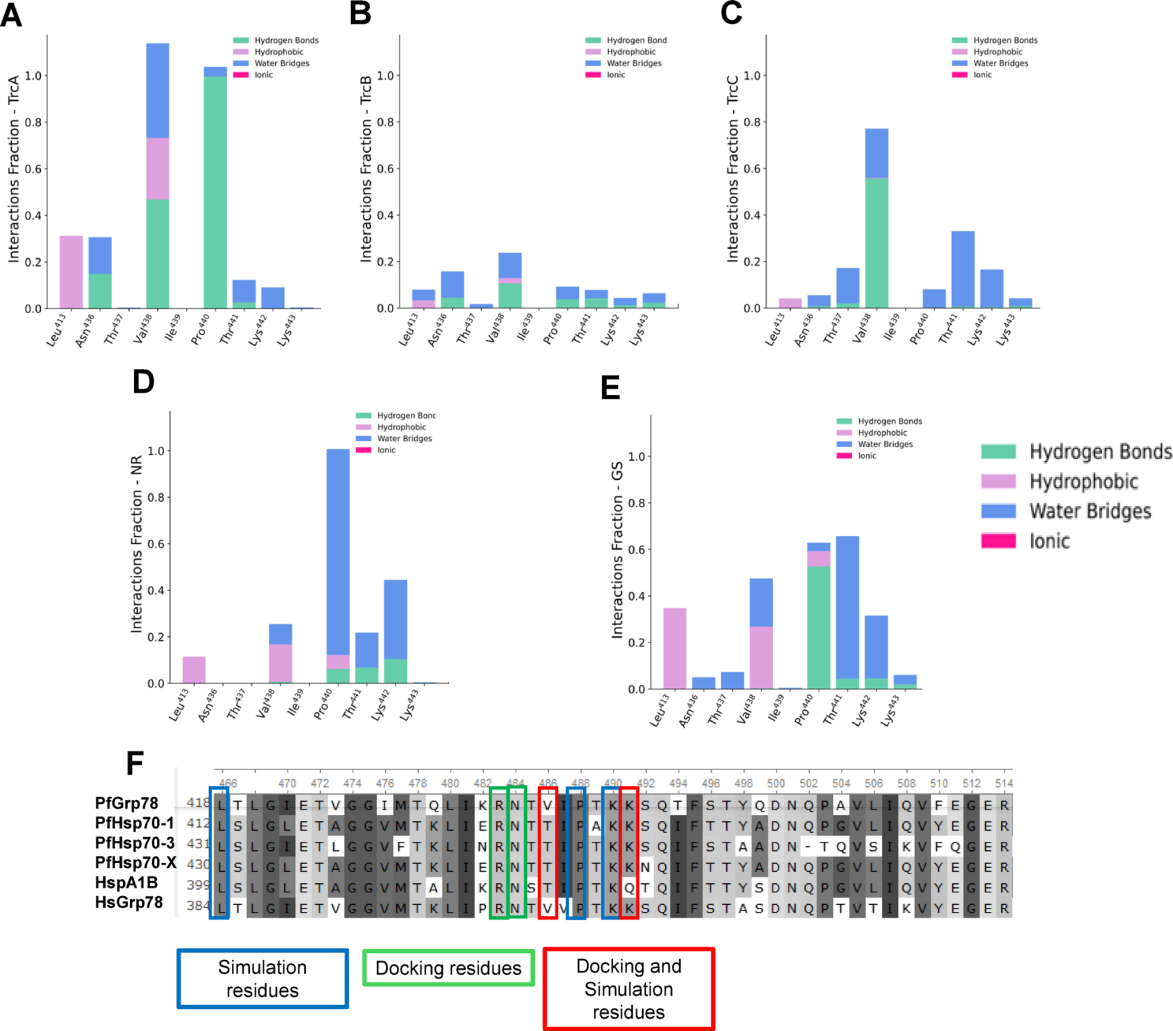
Comparative analysis of the *Hsp70* residues that bind peptide ligands. **A**–**E** Interactions fraction of the SBD amino acid residues of *Pf*Grp78 with the peptides as identified in MDS with **A** SBD -*Pf*Grp78-TrcA, **B** SBD-*Pf*Grp78-TrcB, **C** SBD of *Pf*Grp78-TrcC, **D** SBD of *Pf*Grp78-GS, **E** SBD of *Pf*Grp78-NR peptide contacts over 1000 ns. **F** Multiple sequence alignment of human and plasmodial Hsp70 with the *Pf*Hsp78 residues that were identified in docking (green), those involved in MD simulation (blue) and those that were identified in both (red)

The intermolecular interactions between the protein and ligand were investigated through the 1000 ns simulation period. The different types of bonds formed that contributed to the stability of the complex include hydrophobic contacts, ionic interaction with hydrogen bonds and water bridges forming the strongest bonds. The interaction fractions suggest that TrcA had more stable interactions with *Pf*Grp78 SBD, which are dominated by hydrogen bonds on Val^438^ and Pro^440^ (Fig. 5A). This was followed by TrcC and NR which had less hydrogen bond interaction fractions. The TrcB and GS complexes with *Pf*Grp78, on the other hand, had the lowest hydrogen bonding interaction fractions which resulted in weaker association facilitated through water bridges (Fig. 5B–E). The amino acid residue, Asn^436^ of the *Pf*Grp78 SBD was involved in the interaction with TrcA in both molecular docking and MD simulation. In addition, Pro^440^ and Val^438^, with Thr^441^ and Asn^436^ were predominant *Pf*Grp78 SBD residues that are hydrogen bonding partners in both TrcA and GS binding. In general, TrcA has substantially high interaction fractions, and its structural analogues (TrcB and TrcC) have fewer interaction fractions across trajectory frames. The multiple sequence alignment was used to highlight the sequence conservation of residues implicated in molecular docking and MD simulations for *Pf*Grp78 and human Hsp70 homologs (Fig. 5F). The alignment shows that *Pf*Grp78 VIP motif residues was the main interaction site with the peptides during both MD simulations and docking studies. This suggests that this motif could be central to the predicted binding preference of *Pf*Grp78.

**Fig. 6:**
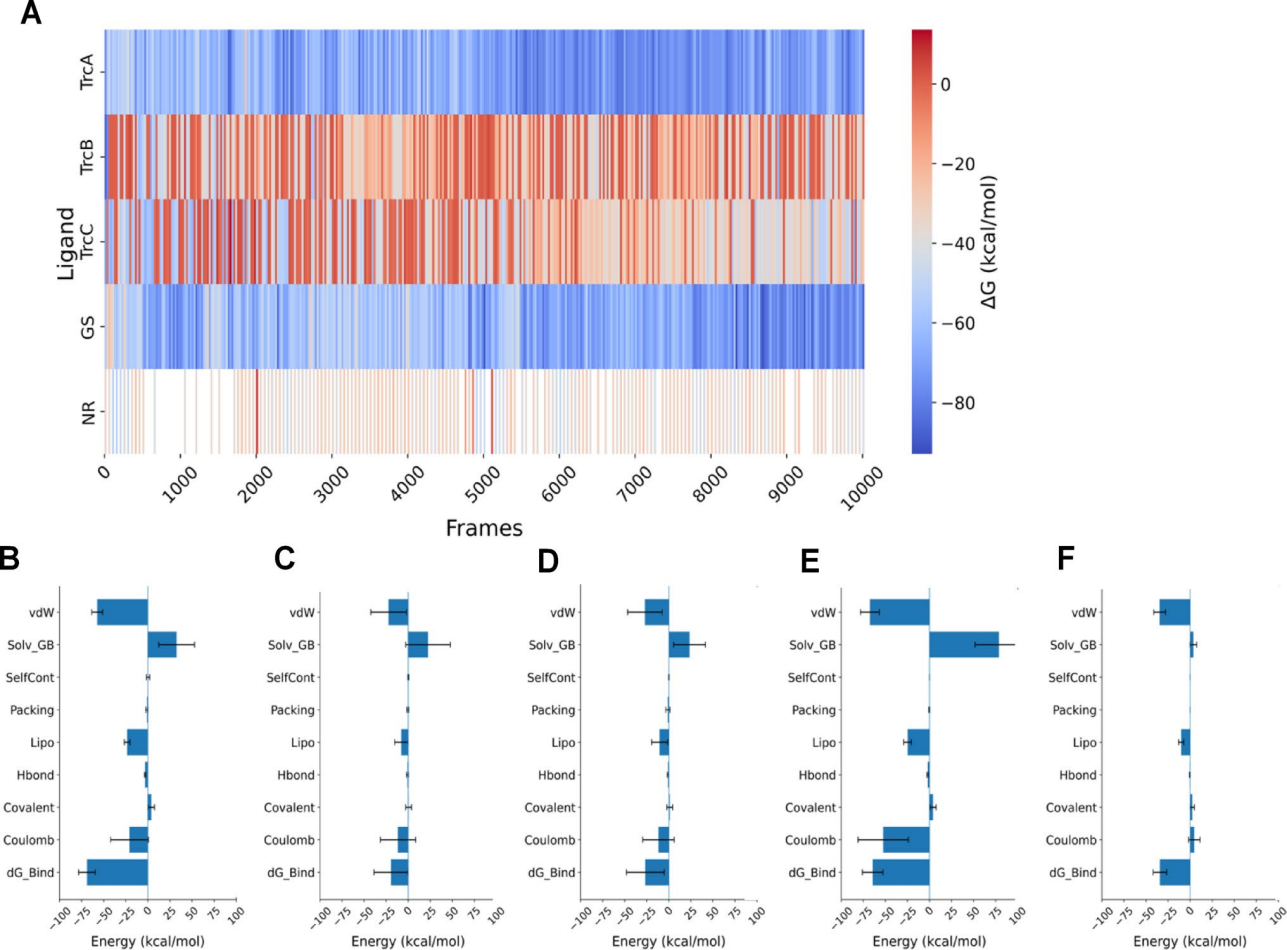
MM–GBSA binding free-energy profiles for *Pf*Grp78–peptide complexes. **A** Heatmap showing the MM–GBSA binding free energy (ΔG, kcal mol⁻^1^) for each peptide ligand (NR, TrcA, TrcB, TrcC, and GS) in complex with PfGrp78, calculated across 10,000 frames sampled at 25-frame intervals from the molecular dynamics trajectories. The colour scale (red to dark blue) reflects progressively more favourable binding free energies. TrcA and GS exhibit consistently more negative ΔG values, indicative of more stable and energetically favourable interactions with PfGrp78 relative to the NR control. **B**–**F** Decomposition of energetic contributions to the total binding free energy (ΔG_bind) for *Pf*Grp78 in complex with **B** TrcA, **C** TrcB, **D** TrcC, **E** GS and **F** NR. Individual components include Coulombic interactions, covalent terms, van der Waals (vdW) energies, lipophilic contributions (Lipo), generalized Born electrostatic solvation (SolvGB), hydrogen-bonding terms (Hbond), π–π packing corrections (Packing), and self-contact corrections (SelfCont). These profiles highlight the distinct energetic signatures underpinning ligand engagement with *Pf*Grp78

The binding free energy (ΔG_bind) analysis using the MM-GBSA method revealed that TrcA and GS peptides bind more favourably to *Pf*Grp78 with ΔG_bind of − 68.82 kcal/mol and − 64.44 kcal/mol respectively compared to the binding of NR (− 34.21 kcal/mol), TrcB (− 19.48 kcal/mol) and TrcC (− 26.64 kcal/mol) (Table 3 and Fig. 6A). It should be noted that TrcB and TrcC registered high fluctuations variability during the sampled frames, with slightly low binding energies during the first 250 ns corresponding to 2500 frames and there after it had higher binding energies which resulted in high standard deviation values for the whole simulation period of 1000 ns (Table 3). The low variability of TrcA and GS values suggest that TrcA and GS form more stable and energetically favourable complexes with *Pf*Grp78, which may reflect a higher degree of intermolecular interactions such as hydrogen bonding and hydrophobic contacts within the binding cavity. The relatively lower binding energy of NR over the 10 000 frames corresponding to 1 µs indicates fewer or weaker stabilising contacts, consistent with its observed behaviour during the MD simulation. On further analysis of the contributions of different interaction properties, it was observed that Van der Waals energy contributed to better binding across all the peptides (Fig. 6B–F). The generalized Born electrostatic solvation energy had the highest impact of reducing the ΔG_bind in GS when compared to the other peptides. This can be interpreted as GS required the most energy to be transferred from vacuum into water as a solvent in a way as a measure of how the solvent the charges on GS. To counteract this, GS had the highest negative Coulomb energy which stabilised the electrostatic interactions with *Pf*Grp78. As expected, the NR peptide did not have major energy contributions apart from Van der Waals energy (Fig. 6F). Taken together, these findings further support the preference of *Pf*Grp78 to bind to TrcA and GS over the other peptides.

**Fig. 7:**
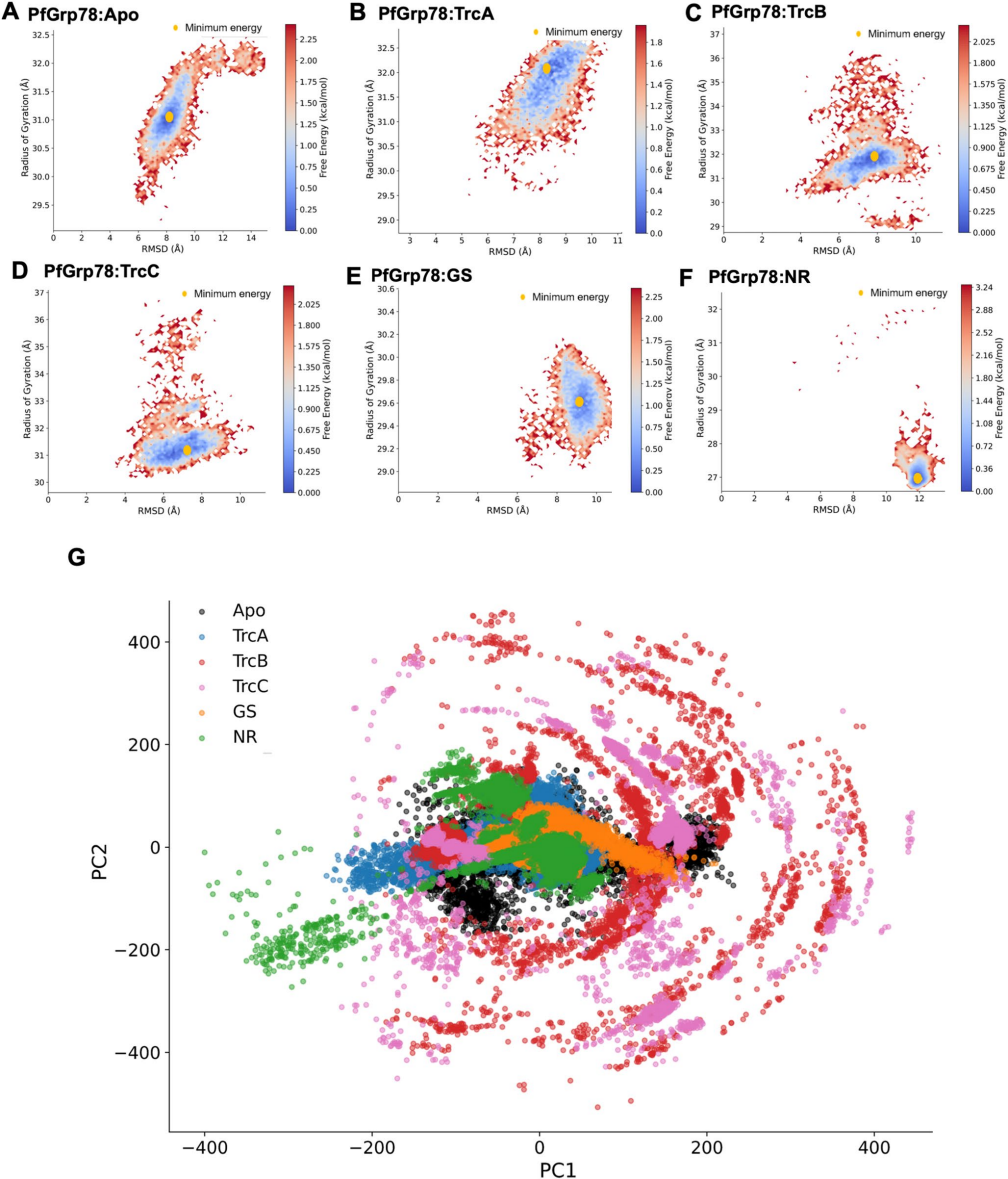
Free‑energy landscapes and principal component analysis of *Pf*Grp78 conformational states. **A**–**F** Free‑energy landscapes (FELs) of *Pf*Grp78 projected onto backbone RMSD and radius of gyration (Rg) for each Apo protein—**A** Apo, and the peptide‑bound complex **B** TrcA, **C** TrcB, **D** TrcC, **E** GS—and **F** NR. Colour gradients represent relative free energy (ΔG), with blue indicating low‑energy basins corresponding to stable conformational states, and red indicating higher‑energy, less populated transition regions. The distinct basin topologies reflect ligand‑specific modulation of *Pf*Grp78’s conformational ensemble. **G** Two‑dimensional projection of molecular dynamics trajectories onto the first two principal components (PC1 and PC2), illustrating the dominant collective motions sampled by Apo *Pf*Grp78 (black) and the peptide‑bound complexes: NR (green), TrcA (blue), TrcB (red), TrcC (pink), and GS (orange). The clustering patterns highlight ligand‑dependent shifts in conformational space, revealing how different peptides remodel the global dynamics of the *Pf*Grp78 substrate‑binding domain

The analysis of RMSD-Rg free energy landscapes to understand conformational states that are mostly thermodynamical favourable revealed distinct conformational signatures for each *Pf*Grp78 system (Fig. 7A). The apo protein exhibits a broad, shallow energy basin spanning RMSD of around 6.5–10.0 Å and Rg 30–32 Å, indicative of inherent structural flexibility required for its chaperone open and closed conformation during substrate binding events. On binding to TrcA the protein-compound complex moves into a conformation that has a higher radius of gyration suggestive of expanded /relaxed conformation (Fig. 7B). The association with GS shifts the complex into a high RMSD suggestive of structural rearrangement or more unfolded conformation (Fig. 7C). However, when bound to either TrcB or TrcC the protein-compound complex shifts to a conformation that has low Rg suggestive of a more compact protein albeit with some flexible ensembles observed (Fig. 7D–E). Intriguingly, when bound to NR peptide, *Pf*Grp78 shifts into a unique conformation with low Rg suggestive of compact and high RMSD (Fig. 7F). This suggests that the presence of NR cause the protein to rearrange into a conformation that is dramatically different from the peptides which could be due to the folding cycle of the chaperone and into which it could not assume when bound to TrcA.

To further elucidate the impact of peptide binding on the global dynamics and conformational landscape of *Pf*Grp78, principal component analysis (PCA) was performed on the molecular dynamics trajectories. The collective motions of the protein in each system were projected onto the first two principal components of eigenvectors of the covariance matrix (PC1 and PC2), which capture the most dominant large-scale motions. The resulting 2D projection plot (Fig. 7G) reveals conformational sampling for each system. The *Pf*Grp78-Apo (black) samples a wide region of the conformational space, suggesting the protein's inherent flexibility and dynamic nature. The bound state *Pf*Grp78 occupied defined regions of the conformational space. The *Pf*Grp78-TrcA (blue) and the *Pf*Grp78-GS (orange) systems are clustered into more compact regions, suggesting a maintenance or a fair reduction in the overall structural flexibility of *Pf*Grp78 upon binding to these two peptides. On the contrary, the *Pf*Grp78-NR (green) complex populates a different area of the conformational space, shifted away from the Apo and other bound states, suggesting it induces a different conformational state or set of collective motions in the chaperone. The *Pf*Grp78-TrcB (red) and *Pf*Grp78-TrcC (sky blue) complexes further sampled a wider area without finely defined conformational space sampling suggestive of flexibility.

To visualize the structural impact of peptide binding, the minimum energy conformer for each complex, as identified from the free energy landscapes analysis, was superimposed onto the α-carbon atoms of the *Pf*Grp78-Apo reference structure (Supplementary Figure S13). The superposition of the *Pf*Grp78-NR complex (green) shows a substantial structural rearrangement, evidenced by a high RMSD of 13.89 Å (Supplementary Figure S13A). This value is consistent with a large global rearrangement rather than the stabilization of a pre-existing state, in agreement with the PCA results, which showed the *Pf*Grp78-NR complex occupying a distinct region of conformational space. In contrast, the superposition of the most stable *Pf*Grp78-TrcA complex (blue) with the apo structure (black) results in a low RMSD of 5.56 Å (Supplementary Figure S13B). This structural similarity indicates that TrcA, the most potent binder, does not induce a dramatic rearrangement. Instead, it appears to operate by binding to and stabilising a low-energy conformation that is already part of the Apo protein's native ensemble. By locking the chaperone in this state, TrcA may decrease the structural plasticity required for its function, which is consistent with the deep, narrow energy well observed in its free energy landscapes plot. Similarly, the *Pf*Grp78-GS complex (orange) aligns with the Apo structure with an RMSD of 8.16 Å (Supplementary Figure S13C). While this deviation is larger than that of TrcA, it still may point to the stabilisation of a native-like fold. TrcB and TrcC show RMSD of 8.51 Å and 7.084 Å when aligned with the Apo minima structure, albeit with weaker free binding energies with *Pf*Grp78. Both TrcA and GS may achieve their effect by trapping the protein in a specific conformation, thereby inhibiting its dynamic cycle. Taken together, this suggests that the NR peptide forces the chaperone to adopt a globally different and energetically less favourable conformation when compared to both TrcA and GS.

### ADME/T analysis

The ADMET analysis of cyclodecapeptides, TrcA and GS, and linear NR peptide were predicted on Qikprop module of on Schrodinger software. The results show that the peptides were above the allowed *M*_r_ range and violated the Lipinski rule of five [57] with three violations on size, number of hydrogen bond donors and acceptors (Table 4). It has been proposed that as peptide-based drugs do not mainly fit the rule of 5 for small molecule orally administered drugs, they fill the gap between small molecules and biologics [58]. The peptides were within the allowable range for QPlog po/w and Q PlogS. These parameters indicate the low lipophilicity of the peptides which affects their solubility [54]. This means that these peptides have a poor passive transcellular diffusion therefore transport can be mediated through QPPCaco, which involves the uptake of hydrophilic peptides through passive diffusion and therefore prevent the inhibition of hERG channel [59]. In addition, according to the new classification they are within the 95th percentile as described by Santos and colleagues [58], the Mr, logP, hydrogen bond donors and acceptors were within range for oral peptide drugs. Although TrcA and GS were predicted to be soluble in aqueous solutions, their use may need further improvement as peptide-based drugs are known for their instability as they are mostly susceptible to proteolysis [59]. Considering that these are cyclodecapeptides, their cyclic structures have the potential to increase their bioavailability due to a potential longer in vivo stability [60]. Taken together, these indices show the promise of these peptide-based inhibitors as potential scaffold for malaria therapy which may need further improvement to increase their efficacy.

**Table 4:** ADMET properties of TrcA and GS cyclodecapeptides

ADME/T properties											
Peptide	*M*_r_	dipole	SASA	FOSA	FISA	PISA	WPSA	Volume	dnHB	acHB	HOA
TrcA	1348.57	19.27	1430.6	517.03	364.6	549.0	0	3455.4	11	26	1
TrcB	1309.53	11.82	1304.09	506.55	328.86	468.683	0	3256.54	10	25	1
TrcC	1348.57	19.1	1436.71	525.14	363.85	547.72	0	3461	11	25	1
GS	1141.46	8.95	1218.6	811.36	160.5	246.7	0	3035.2	6	22	1
NR	712.89	8.31	1242.4	738.70	503.7	0	0	2348.8	8	16	1

ADME/T properties											
Peptide	QlogPo/w	logS	CIQPlogS	QPlogHERG	QPPCaco	QPlogBB	QPPMDCK	QPlogKp	QPlogKhsa	PSA	ROF5
TrcA	− 1.08	0.64	− 8.36	7.94	0.07	− 5.52	0.27	− 4.53	− 3.16	468.6	3
TrcB	− 1.8	2	− 7.527	9.0	0.18	− 4.75	0.62	− 4.15	− 3.43	442.84	2
TrcC	− 1.05	0.52	− 8.36	7.90	0.076	− 5.54	0.27	− 4.52	− 3.15	470.53	3
GS	0.413	1.122	− 5.33	6.86	4.332	− 2.355	8.120	− 4.278	− 2.39	341.5	3
NR	− 1.76	− 1.883	− 1,79	0.94	0.004	− 7.76	0.010	− 12.4	− 1.83	366.7	3

Qikprop descriptors and the recommended ranges: *M*_r_= relative molar mass (130–725), dipole=computed dipole moment (1.0–12.5), SASA= total solvent accessible surface area (300–1000), FOSA= hydrophobic component of SASA (0–750), FISA= hydrophilic component of SASA (7-330), PISA= carbon pi component of SASA (0–450), WPSA= weakly polar component of SASA (0–175), volume= molecular volume (Å^3^) (500–2000), dnHB= donor hydrogen bonds (0–6), acHB = acceptor hydrogen bonds (2–20), HOA= percentage human oral absorption in GI (low <25% high >85%) (< 25% is poor), logPo/w= log P for octanol/water (− 2–6.5), log S= S for aqueous solubility (− 6.5–0.5), CIQlog= log S conformation-independent (− 6.5–0.5), QPPlogHERG= predicted IC_50_ value for blockage of HERG K + channels (Concern <− 5), QPCaco= predicted gut blood barrier model Caco-2cell permeability in nm/sec (< 25 poor/ >500 great), QPlog BB= log blood/brain partition coefficient B for brain/blood (− 3.0–1.2), QPPMDCK permeability (nm/s) (< 25 poor, >500 excellent), Qlog Kp= log Kp for skin permeability (Kp in cm/h − 8.0/− 1), log Khsa = log khsa serum albumin protein binding (− 1.5–1.5), PSA+ Van der Waal’s polar surface area (7–200), RO5= lipinski rule of 5 violations (maximum is 4)

## Discussion

*Pf*Grp78 is a central regulator of the ER unfolded protein response (UPR) in *P. falciparum*, where it contributes to parasite survival under febrile and drug-induced stress [1, 10]. In this context, selectively targeting *Pf*Grp78 could enhance proteostasis collapse and sensitise the parasite to ER stress–inducing conditions. Although *Pf*Grp78 shares high overall sequence identity with its human orthologue *Hs*Grp78, comparative analyses in this study reveal substrate-binding-domain (SBDβ) features that distinguish it from both human and parasite cytosolic Hsp70s (Fig. 1). Sequence identity between *Pf*Hsp70—1 and *Pf*Grp78 was moderate (58%), whereas the full length *Pf*Grp78 displayed substantially higher identity (84%) to *Hs*Grp78, confirming phylogenetic relatedness but also underscoring the potential for selective pharmacological targeting [11, 61]. The 3D structural superpositions showed conservation of the canonical Hsp70 fold, yet revealed differences within the hydrophobic arch, pocket, and neighbouring loops regions previously implicated in functional divergence of Hsp70s across species [62, 63].

Substrate selectivity is a defining feature of Hsp70 function. Across the family, SBDs are the least conserved domains [61] and accommodate different residue preferences depending on their cellular compartment [23]. In this study, we found that, unlike human Grp78 (*Hs*Grp78), *Pf*Grp78 showed a predicted preference for hydrophobic interactors but limited engagement with aromatic residues (Fig. 3; Supplementary Table S2), suggesting a distinct substrate-binding surface. The ER-resident *Pf*Grp78 thus appears to accommodate peptide motifs different from those preferred by *Hs*Grp78, providing a structural rationale for parasite-specific ligand recognition.

Cyclodecapeptides were evaluated for their predicted interactions with *Pf*Grp78. Docking analyses identified TrcA as the highest scoring ligand, forming multiple hydrogen bonding and hydrophobic contacts with SBDβ residues, including Arg^435^, Thr^437^, Val^438^, and Lys^443^ (Fig. 3; Table 2). Several of these residues are located within regions that differ structurally from related human Hsp70 homologues, suggesting that they may contribute to the observed binding preferences. TrcA and GS are known to exhibit antiplasmodial activity and membrane disruptive properties in previous studies [30–32, 64, 65] The predicted interactions observed here raise the possibility *Pf*Grp78 could represent an additional molecular target associated with their biological activity, however, direct experimental evidence demonstrating *Pf*Grp78 engagement with these peptides is currently lacking.

Previous studies have attributed the bioactivity of tyrocidines to their amphipathic organization and hydrophobic surface properties [46, 64, 65]. In line with these characteristics, energy-decomposition analysis indicated that van der Waals and lipophilic interactions contributed substantially to the predicted binding energetics of TrcA (Fig. 6). Additional electrostatic contributions involving Arg^435^ and Lys^443^ were also observed which potentially enhance binding stability despite potential charge repulsion [64, 65]. Such non-covalent interactions including hydrogen bonds, π–π contacts, and ionic interactions have been reported as important contributors to ligand recognition and binding within Hsp70 family proteins [66]. These results therefore provide a structural basis for the predicted association between TrcA and the *Pf*Grp78 substrate binding domain.

MDS supported the docking analysis by indicating that TrcA and GS formed the comparatively stable complex with *Pf*Grp78 over the simulation period (Fig. 4; Table 3). The apo protein exhibited a broad free energy landscape containing accessible minima (Fig. 6), consistent with conformational heterogeneity during the simulation. In line with conformational plasticity required for ATP-mediated cycling [67–69]. In contrast, TrcA binding was associated with a deeper and narrower free-energy basin (Figs. 6, 7), suggesting reduced exploration of conformational space relative to the apo state. This observation may reflect preferential stabilisation of a restricted subset of *Pf*Grp78 conformations; however, simulations alone cannot establish whether such behaviour translates into altered chaperone activity under physiological conditions.

The MM-GBSA binding free energies reported here represent effective enthalpy-dominated free-energy estimates that do not explicitly include solute configurational entropy contributions. This approach is commonly employed for ranking structurally related ligands because entropy estimation methods, including normal-mode and quasi-harmonic entropy estimates are often associated with substantial uncertainties [70]. Consequently, the calculated binding free energies should be interpreted primarily as relative rather than absolute measures of binding affinity. While entropy was not explicitly incorporated into the MM-GBSA calculations, the free-energy landscape and principal component analyses provide information regarding conformational sampling at the receptor level. The more compact conformational distributions observed for TrcA, and to a lesser extent GS, are consistent with reduced sampling of the PfGrp78 conformational ensemble during simulation time. These results suggest a potential effect of ligand binding on protein dynamics, although experimental studies would be required to determine the functional significance of these observations.

Structural superposition analysis revealed substantial similarities between the TrcA-bound and apo minimum-energy conformers (RMSD = 5.56 Å; Supplementary Figure S13A). Together with the free-energy landscape analysis, these findings suggest that TrcA may favour certain *Pf*Grp78 conformational states without inducing major alterations to the overall protein architecture. GS produced a qualitatively similar pattern, although the effect was less pronounced. In contrast, NR -bound conformer showed greater structural deviation from the apo state (RMSD = 13.89 Å; Supplementary Figure S13C), indicating that alternative conformational states were sampled during the simulation. The principal component analysis (Fig. 7G) further showed that TrcA- and GS-bound systems occupied a more restricted conformational space than TrcB-, TrcC-, and NR-bound systems. Collectively, these observations suggest that different ligands are associated with distinct *Pf*Grp78 dynamic behaviours.

Taken together, the computational analyses identify *Pf*Grp78 as a potentially tractable target for structure guided antimalarial discovery. Our results suggest that TrcA preferentially engages a parasite-specific binding pocket and promotes a low-energy conformational ensemble with altered behaviour relative to the apo state. These insights provide hypotheses regarding how cyclodecapeptides may interact with *Pf*Grp78 and highlight residues that may warrant further investigation. However, the proposed mechanism remains predictive and requires experimental validation through direct binding measurements, structural studies, targeted mutagenesis of the SBDβ binding cleft, and parasite-based functional assays. Such studies will be necessary to determine whether the computationally predicted interactions contribute to the antiplasmodial activities of these compounds and assess the therapeutic potential of inhibitors directed at the *Pf*Grp78-dependent UPR pathways.

### Limitations of the study

This study relies entirely on computational approaches, including molecular docking, molecular dynamics simulations, and MM-GBSA analyses. While these methods are useful for identifying and prioritising potential *Pf*Grp78 ligands, they provide predictive rather than experimental evidence of binding and biological activity. Furthermore, the simulations do not account for factors such as cellular uptake, pharmacokinetics, toxicity, or off-target effects. Although TrcA and GS showed favourable predicted interactions with *Pf*Grp78, direct experimental evidence of target engagement and modulation of *Pf*Grp78-dependent stress responses in *P. falciparum* remains limited. Accordingly, the proposed mechanism should be regarded as a testable hypothesis requiring validation through biophysical, biochemical, and parasite-based studies.

### Assession codes

Uniprot identifiers: *Plasmodium falciparum* Endoplasmic reticulum chaperone BIP: Q8I2X4, *Plasmodium falciparum* heat shock protein 70: P11144, *Plasmodium falciparum* heat shock protein 70: Q8II24, *Plasmodium falciparum* heat shock protein 70: K7NTP5, *Homo sapiens* heat shock 70 kDa protein 1A: P0DMV8, *Homo sapiens* heat shock 70 kDa protein 1B: P0DMV9, *Homo sapiens* heat shock 70 kDa protein 1-like: P34931, *Homo sapiens* heat shock 70 kDa protein 2: P54652, *Homo sapiens* glucose regulated 78 kDa protein/Endoplasmic reticulum chaperone BiP*:* P11021, *Homo sapiens* heat shock 70 kDa protein 6: P17066, *Homo sapiens* heat shock 71 kDa protein: P11142, *Homo sapiens* Stress-70 protein, mitochondrial: P38646.

## Supplementary Information

Below is the link to the electronic supplementary material.

Supplementary Material 1

Supplementary Material 2

## Data Availability

All data presented in the current study are contained within the manuscript/ Supplementary Materials and accessible from the corresponding author including simulation videos upon request.
