# Supplementary material for: In silico identification of selective cyclodecapeptide inhibitors targeting Plasmodium falciparum Grp78 chaperone
Source: J Comput Aided Mol Des. 2026 Aug 1;40(1):192. doi: 10.1007/s10822-026-00907-1 (PMC13428706; doi:10.1007/s10822-026-00907-1)
Supplement: Supplementary file 2 — Supplementary Material 2 [file 10822_2026_907_MOESM2_ESM.docx]

***In silico* identification of selective peptide inhibitors targeting *Plasmodium falciparum* Grp78 chaperone**

Wendy Mthembu^1^, Adeshina Odugbemi^1^, Florence Lisa Muzenda^1^, Marina Rautenbach^1^, Tawanda Zininga^1^*

^1^Department of Biochemistry, Stellenbosch University, Stellenbosch, 7600, South Africa

*Corresponding author: [tzininga@sun.ac.za](mailto:tzininga@sun.ac.za)

**Supplementary Information**

**Table S1 – page S2**

**Table S2 – page S2**

**Table S3 – page S3**

**Table S4 - page S4**

**Table S5 – separate Excel sheet**

**Figure S1 - page S6**

**Figure S2 - page S7**

**Figure S3 - page S8**

**Figure S4 - page S9**

**Figure S5 - page S10**

**Figure S6 - page S11**

**Figure S7 - page S12**

**Figure S8 - page S13**

**Figure S9 - page S14**

**Figure S10 - page S15**

**Figure S11 - page S16**

**Figure S12 - page S17**

**Figure S13 - page S18**

**Table S1. Hsp70 predicted druggability in SiteMap**

| **Hsp70 Homologs** | **Dscore prediction (kcal)** |
| --- | --- |
| *Pf*Grp78 | 1.055 |
| *Pf*Hsp70-*1* | 1.011 |
| *PfHsp70-3* | 1.033 |
| *Hs*Hsp70 | 0.973 |
| *Hs*Grp78 | 0.998 |

**Table S2. Hsp70 homologs and Molecular docking analysis**

| Hsp70 homolog |  |  | | Docking scores (kcal/mol) | | |
| --- | --- | --- | --- | --- | --- | --- |
|  | TrcA | TrcB | TrcC | | GS | NR |
| HspA1A (P0DMV8) | NB | NB | NB | | NB | -8.749 |
| HspA1B (HsHsp70, P0DMV9) | -5.188 | NB | -3.750 | | -4.960 | -8.127 |
| HspA1L P34931 | NB | NB | NB | | NB | -7.612 |
| HspA2 (P54652) | NB | NB | NB | | NB | -9.291 |
| HspA5 (HsGrp78, P11021) | NB | NB | NB | | NB | -5.666 |
| HspA6 (P17066) | NB | NB | NB | | NB | -7.255 |
| HspA8 | NB | NB | NB | | NB | -7.642 |
| HspA9 | NB | NB | NB | | NB | -9.266 |
| *Pf*Grp78 | -7.434 | -6.39 | -7.23 | | -6.62 | -7.521 |
| *Pf*Hsp70-1 | NB | -3.097 | NB | | -4.33 | -7.309 |
| *Pf*Hsp70-3 | -7.069 | -3.389 | -6.190 | | -5.902 | -7.833 |
| *Pf*Hsp70X | NB | NB | NB | | NB | -7.256 |
| DnaK (4B9Q) | -5.616 | -5.462 | -4.988 | | -4.887 | -7.555 |

NB**:** no binding predicted, Docking score: kcal/mol

**Table S3. Analysis of the effect of PfGrp78 SBD mutations on peptide interaction**

| **PfGrp78 Mutation** | **Docking to GS** | **Docking to TrcA** | **Docking to NR peptide** |
| --- | --- | --- | --- |
| PfGrp78 (V^438^-P^440^) | -6.617 Kcal/mol | -7.434 Kcal/mol | -7.521 Kcal/mol |
| PfGrp78 (V^438^L) | NB | NB | -7.900 Kcal/mol |
| PfGrp78 (I^439^P) | NB | NB | -6.904 Kcal/mol |
| PfGrp78(P^440^I) | NB | NB | -6.924 Kcal/mol |
| PfGrp78 (L^438^,P^439^,I^440^) | NB | NB | -7.130 Kcal/mol |

NB**:** no binding predicted

| Table S4. Simulation quality analysis the protein and peptide complexSystem simulated | Parameter | Average | Standard deviation | Slope (ps^-1^) |
| --- | --- | --- | --- | --- |
| *Pf*Grp78-Apo | Total energy (kcal/mol) | -285514.00 | 149.080 | -0.001 |
|  | Potential energy (kcal/mol) | -348941.775 | 130.385 | -0.001 |
|  | Temperature (K) | 298.709 | 0.432 | 0.000 |
|  | Pressure (bar) | 0.909 | 29.895 | -0.000 |
|  | Volume (A^3)^ | 1041407.641 | 609.190 | 0.001 |
| *Pf*Grp78-TrcA | Total energy (kcal/mol) | -266440.561 | 146.108 | -0.002 |
|  | Potential energy (kcal/mol) | -325935.282 | 129.499 | -0.002 |
|  | Temperature (K) | 298.704 | 0.440 | 0.000 |
|  | Pressure (bar) | 0.848 | 31.700 | -0.000 |
|  | Volume (A^3)^ | 973670.628 | 614.821 | -0.003 |
| *Pf*Grp78-GS | Total energy (kcal/mol) | -268545.199 | 156.433 | -0.002 |
|  | Potential energy (kcal/mol) | -328395.485 | 139.834 | 0.000 |
|  | Temperature (K) | 298.708 | 0.437 | 0.000 |
|  | Pressure (bar) | 1.119 | 31.510 | -0.000 |
|  | Volume (A^3)^ | 979424.955 | 614.223 | -0.003 |
| *Pf*Grp78-NR | Total energy (kcal/mol) | -266342.974 | 150.667 | -0.002 |
|  | Potential energy (kcal/mol) | -325773.019 | 133.813 | -0.002 |
|  | Temperature (K) | 298.705 | 0.439 | 0.000 |
|  | Pressure (bar) | 1.366 | 31.152 | 0.000 |
|  | Volume (A^3)^ | 972610.778 | 614.654 | -0.002 |
| *Hs*HspA1B-GS | Total energy (kcal/mol) | -254707.354 | 143.747 | -0.001 |
|  | Potential energy (kcal/mol) | -315417.436 | 126.626 | -0.001 |
|  | Temperature (K) | 308.684 | 0.455 | 0 |
|  | Pressure (bar) | 0.943 | 31.929 | 0 |
|  | Volume (A^3)^ | 972206.7 | 630.237 | -0.004 |
| *Hs*HspA1B-TrcA | Total energy (kcal/mol) | -259028.451 | 148.372 | -0.002 |
|  | Potential energy (kcal/mol) | -320657.224 | 131.601 | -0.002 |
|  | Temperature (K) | 308.691 | 0.451 | 0 |
|  | Pressure (bar) | 1.311 | 31.439 | 0 |
|  | Volume (A^3)^ | 987434.038 | 616.55 | -0.003 |
| *Pf*Hsp70-1-GS | Total energy (kcal/mol) | -323079.806 | 360.926 | 0 |
|  | Potential energy (kcal/mol) | -399540.6 | 295.59 | 0 |
|  | Temperature (K) | 308.672 | 0.826 | 0 |
|  | Pressure (bar) | 0.952 | 71.135 | 0 |
|  | Volume (A^3)^ | 1232951.78 | 1600.301 | 0.002 |
| *Pf*Hsp70-3-GS | Total energy (kcal/mol) | -387356.249 | 334.973 | -0.002 |
|  | Potential energy (kcal/mol) | -478413.675 | 276.661 | -0.002 |
|  | Temperature (K) | 308.693 | 0.63 | 0 |
|  | Pressure (bar) | 1.169 | 54.358 | 0 |
|  | Volume (A^3)^ | 1475518.815 | 1488.751 | -0.01 |

**Figure S1. Flowchart characterizing the peptide as potential binders of Hsp70s.** The proteins were first aligned to identify unique motifs and then the active sites before the subsequent docking and molecular dynamics simulations.


**Figure S2. The 3D and 2D analysis of *Pf*Grp78 -peptide docking.** The first panel represents the *Pf*Grp78 SBD peptide bound, the second panel shows the surface of the binding cavity and the orientation of the peptide, the third panel represents the 3D structure of the binding complexes and finally the 2D showing the bonding types and interacting residues. **A** *Pf*Grp78-TrcA, **B**. *Pf*Grp78-TrcB, **C.** *Pf*Grp78-TrcC, **D.** *Pf*Grp78-GS, **E.** *Pf*Grp78-NR. The nature of the binding interactions was mainly comprised of hydrogen bonds and salt bridges as shown by the coloured lines between protein-ligand complexes. The predicted docking poses were generated using Schrodinger Maestro 2022.

**Figure S3. The Hsp70 – peptide molecular docking.** The human Hsp70 homolog was docked with GS and TrcA. A) HSPA1B-GS, B) HSPA1B-TrcC and C) HSPA1B-NR. The docking position is shown in cartoon and the binding pocket together with the 2-D docking mode with residue and type of bonds shown. The docking scores are shown.

**Figure S4. The *Pf*Hsp70-1 – peptide molecular docking.** The *Pf*Hsp70-1 was docked with peptides A) *Pf*Hsp70-1+TrcB, B) *Pf*Hsp70-1 -GS, C) *Pf*Hsp70-1+NR. The docking position is shown in cartoon and the binding pocket together with the 2-D docking mode with residue and type of bonds shown. The docking scores are shown.

**Figure S5. The *Pf*Hsp70-3 – peptide molecular docking.** The *Pf*Hsp70-3 homologs was docked with peptides. A) *Pf*Hsp70-3 +TrcA, B) *Pf*Hsp70-3+TrcB, C) *Pf*Hsp70-3+TrcC, D) *Pf*Hsp70-3+GS and E) *Pf*Hsp70-3-TrcA The docking position is shown in cartoon and the binding pocket together with the 2-D docking mode with residue and type of bonds shown. The docking scores are shown

**Figure S6. *Pf*Grp78 mutants and NR peptide interactions**. Conservative amino acid substitution was performed on the *Pf*Grp78 VIP substrate binding motifs, whereby each amino acid was alternated for another amino acid and docking analysis was performed. Cyclic decapeptide TrcA and GS did not exhibit any binding and thus were not incorporated in the analysis. **A)** Substitution of Val^438^ with Leu^438^, **B)** Substitution of Ile^439^ with Pro^439^ **C)** Substitution of Pro^440^ with Ile^440^, **D)** Replacement of the VIP^438-440^ motif with LPI^438-440^ motif.

**Figure S7. The *Pf*Grp78-ligand stabilities during MD simulation**. A: The Rg of Cα atoms. B: The RMSD plot of *Pf*Grp78 Cα atoms and over a simulation period of 100 ns. C: *Pf*Grp78 RMSF of apo and in complex with TrcA, TrcB, TrcC, GS and NR peptide. D: RMSF of ligands with respect to protein shows the stability of the peptides with respect to the *Pf*Grp78-binding site.

**Figure S8. Convergence of the *Pf*Grp78 production trajectories.** Cumulative (running) averages, from t = 0, of (A) the backbone Cα-RMSD, (B) the radius of gyration, and (C) the MM-GBSA binding free energy (ΔG_bind) over the 1 µs simulations for apo PfGrp78 and the *Pf*Grp78-peptide complexes. Each curve flattens to a stable plateau, and in (C) the ligand ranking is established and maintained from ~400 ns onward, indicating that the time-averaged values in Table 3 reflect converged sampling. ΔG_bind is not applicable in this context for the apo system.


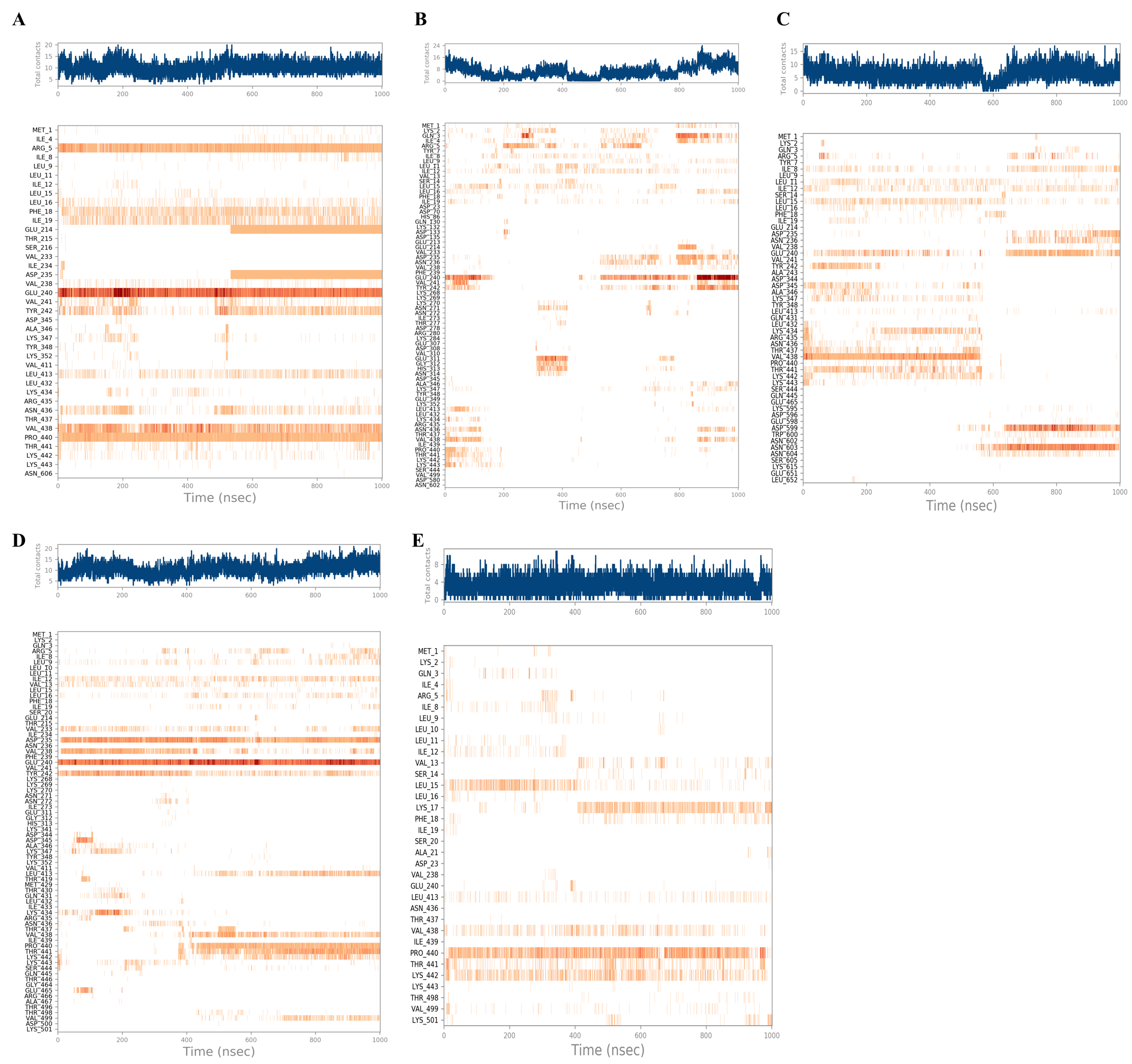


**Figure S9**. Plot of protein-peptide interaction and timeline of the total number of contacts. (A) P*f*Grp78-TrcA, (B) P*f*Grp78-TrcB, (C) P*f*Grp78-TrcC, (D) P*f*Grp78-GS, (E) P*f*Grp78-NR. Images were obtained from the simulation interaction diagram in Maestro v13.1

**Figure S10. The MD simulation *Hs*HspA1B interactions with GS and TrcA over simulation period of 100 ns**. A: The RMSD plot of *Hs*HspA1B Cα atoms (protein RMSD) and GS (ligand RMSD) in complex. B:  *Hs*HspA1B RMSF in complex with GS (yellow block indicates the SBDβ). Residues that interact with the ligand are marked with green vertical bars. C: Interaction fractions of *Hs*HspA1B residues with GS. D: The RMSD plot of *Hs*HspA1B Cα atoms (protein RMSD) and TrcA (ligand RMSD) in complex. B:  *Hs*HspA1B RMSF in complex with TrcA. Residues that interact with the ligand are marked with green vertical bars. C: Interaction fractions of *Hs*HspA1B residues with TrcA.

**Figure S11. The MD simulation of *Pf*Hsp70-1 and *Pf*Hsp70-3 interactions with GS over simulation period of 100 ns**. A) The RMSD plot of *Pf*Hsp70-1 Cα atoms (protein RMSD) and GS (ligand RMSD) in complex. B)  *Pf*Hsp70-1 RMSF in complex with GS (yellow block indicates the SBDβ). Residues that interact with the ligand are marked with green vertical bars. C) Interaction fractions of *Pf*Hsp70-1 residues with GS. D) The RMSD plot of *Pf*Hsp70-3 Cα atoms (protein RMSD) and GS (ligand RMSD) in complex. E)  *Pf*Hsp70-3 RMSF in complex with GS. Residues that interact with the ligand are marked with green vertical bars. F) Interaction fractions of *Pf*Hsp70-3 residues with GS.

**Figure S12:** The Radius of Gyration of protein Cα atoms in the complexes *Hs*HspA1B: TrcA, *Hs*HspA1B:GS, *Pf*Hsp70-1:GS, and *Pf*Hsp70-3:GS

**Figure S13. Structural superposition of energy-minima conformations from PCA analysis.** Comparison of bound state *Pf*Grp78 structures with the apo reference (black), showing conformational changes in low-energy states identified from PCA free energy landscapes. Structures were aligned on Cα atoms, with RMSD values quantifying deviations from *Pf*Grp78-Apo (black). (A) Alignment with *Pf*Grp78-NR (green), (B) *Pf*Grp78-TrcA (blue), *Pf*Grp78-TrcB (red), *Pf*Grp78-TrcC (purple) and (C) *Pf*Grp78-GS (orange).
